# Nitrogen deposition further increases *Ambrosia trifida* root exudate invasiveness under global warming

**DOI:** 10.1007/s10661-023-11380-w

**Published:** 2023-05-30

**Authors:** Ke Xu, Xinyue Liu, Changxin Zhao, Qingmin Pan, Xiaoxing Chen, Ning Jiang, Cuiping Du, Yufeng Xu, Meini Shao, Bo Qu

**Affiliations:** 1 Liaoning Key Laboratory of Biological Invasions and Global Changes, Shenyang Agricultural University, Shenyang, China; 2 Liaoning Panjin Wetland Ecosystem National Observation and Research Station, Shenyang, 110866 China

**Keywords:** *Ambrosia trifida*, Climate warming, Nitrogen deposition, Plant invasion, Widely targeted metabolomes, Root exudates

## Abstract

Invasive plants can change the soil ecological environment in the invasion area to adapt to their growth and reproduction through root exudates. Root exudates are the most direct manifestation of plant responses to external environmental changes, but there is a lack of studies on root exudates of invasive plants in the context of inevitable global warming and nitrogen deposition. In this research, we used widely targeted metabolomics to investigate *Ambrosia trifida* root exudates during seedling and maturity under warming and nitrogen deposition to reveal the possible mechanisms of *A. trifida* adaptation to climate change. The results showed that the organic acids increased under warming condition but decreased after nitrogen addition in the seedling stage. Phenolic acids increased greatly after nitrogen addition in the mature stage. Most phenolic acids were annotated in the phenylpropane metabolic pathway and tyrosine metabolism. Therefore, nitrogen deposition may increase the adaptability of *A. trifida* through root exudates, making it more invasive under global warming. The results provide new ideas for preventing and controlling the invasion of *A. trifida* under climate change.

**Supplementary Information:**

The online version contains supplementary material available at 10.1007/s10661-023-11380-w.

## Introduction

Climate change and biological invasion are the main problems of environmental protection (Wu et al., 2016). Mostly speaking, climate change will aggravate biological invasions (Thomas et al., 2004; Ward & Masters, 2007; Kiritani, 2011). Based on the invasibility of new habitat environments, hypotheses such as the “resource opportunity hypothesis” and the “empty niche hypothesis” have been proposed (Mack, 1996; Davis et al., 2000). These hypotheses argue that available environmental resources at large spatial scales are the key determinants of ecosystem invasibility. Once communities in new habitats have the necessary ecological resources (including nutrients, light, water, soil nutrients) for invasive species, and most of these ecological resources are not effectively used by native species, they provide a possible space for invasion by alien species (Lamarque et al., 2011). The global average temperature increased by 0.85°C in the twentieth century, and the IPCC predicted that it would rise by another 2.6–4.8°C and at least 0.3–1.7°C in the twenty-first century (IPCC, 2007). And the latest report showed that the global average temperature had increased by 1.09 ℃ (IPCC, 2021). Global warming may promote plant invasion by increasing the growth and distribution of invasive plants (Lu et al., 2013; Walther et al., 2009). In theory, warming is expected to make it more likely for invasive plants to effectively spread from the warmer south to the northern hemisphere while also limiting their spread from the cooler north to the southern hemisphere (Koncki & Aronson, 2015). The continued increase in atmospheric nitrogen deposition is an environmental problem that has emerged in recent years and has attracted much attention (Vitousek et al., 1997). We must also take into account how invasive plants may react to nitrogen deposition under a warming climate, which is another component of global environmental change. Nitrogen deposition is expected to be at high levels globally and to increase substantially in Asia (Bobbink et al., 2010; Lamarque et al., 2010). As the most limiting factor for plant growth, higher N supply is more beneficial to exotic invaders than native species under nitrogen deposition (He et al., 2012). However, it is still unclear how invasive plants behave under warming and nitrogen deposition.

Successful invasion soil mechanisms include the production of allelopathic compounds (Callaway & Aschehoug, 2000), alteration of the soil microbial communities (Belnap & Phillips, 2001), and changes in soil nutrient cycling (Levine et al., 2003). Root exudates are significant part in the invasion of exotic plants. Invasive plants can inhibit seed germination of native plants (Pinzone et al., 2018), promote symbiosis with mycorrhizal fungi (Yuan et al., 2014), and improve soil nutrient cycling through root secretions (Hättenschwiler et al., 2000). Extensive literature demonstrates adaptive changes in root exudates under global warming. A study on *Abies faxoniana* found that warming had a significant effect on the relative content of major compounds, with a significant increase in the relative content of phenolic acids, along with a significant increase in soil polyphenol oxidase activity (Qiao et al., 2014); warming increased the secretion of antibacterial chemicals from plant roots, such as organic acids (Wu & Yu, 2019). N enrichment increased *Robinia pseudoacacia* inputs of organic C by 1.5 times (Uselman et al., 2000). It had also been found that high N treatment (100 mg · L^−1^) resulted in a significant reduction in root exudate C input of *Picea asperata* (Aitkenhead-Peterson and Kalbitz, 2010). At present, the research on root exudates of invasive plants mainly focuses on allelopathy (Uddin & Robinson, 2017; Kato-Noguchi, 2020) and its impact on soil microbial activity (Stanek et al., 2021; Stefanowicz et al., 2021), rarely considering changes in global climate. It is not known whether the root exudates of invasive plants, which are mediators of plant-soil material exchange, will significantly change their types and contents under climate change. The variations may give an already highly competitive invasive plant an additional ability in competitive advantage.

*Ambrosia trifida* L. belongs to the Compositae family and is an annual herbaceous plant (Bassett & Crompton, 1982). The plant is native to North America and is a quarantine or noxious weed in many countries, relying mainly on seed dispersal in various means, including human and animal activities and transportation (Hovick et al., 2018). Studies on the dispersal mechanisms of invasive populations of *A. trifida* had shown that it has high genetic diversity and environmental adaptability. By competing with native plants for nutrients, water, and other resources as well as through allelopathy, *A. trifida* could stunt their growth (Kong, 2010). Most studies on *A. trifida* had focused on above-ground parts. However, there are few studies conducted on belowground parts. To effectively prevent and control invasion by invasive plants in the future, it is crucial to comprehend the alterations in root exudates of invasive plants under the influence of climate change, specifically the rise in soil nutrient levels due to increased nitrogen deposition.

We hypothesized that (1) notable changes in the composition and content of *A. trifida* root exudates under warming and significant increases in specific compounds such as phenolic or organic acids could be seen, and (2) after nitrogen addition, root exudates may change differently in different periods. To test these hypotheses, experiments were conducted using widely targeted metabolomics to detect root exudate species and contents, to investigate the patterns of root exudates change under climate change, and to reveal the possible physiological mechanisms. The results help us to further understand the adaptation mechanism of *A. trifida* under climate change and provide a theoretical basis for controlling its spread.

## Materials and methods

### Plant materials and culture methods

The experimental site was arranged in the North Mountain Teaching and Research Base of Shenyang Agricultural University, Shenyang, Liaoning Province (41°49′ N, 123°34′ E). The area has a temperate continental monsoon climate with an altitude of 50 m, four distinct seasons, sufficient sunshine, an average annual temperature of 6~11 °C, an average precipitation of 750~850 mm, and a frost-free period of about 150 days.

The experiment was divided into three groups: control treatment, warming treatment (W), and warming and nitrogen addition treatment (WN). Thirty pots were planted in each treatment. We used sandy soil from Aer Town, Zhangwu Mongolian Autonomous County, Fuxin City, Liaoning Province, with an average pH of 7.18, organic matter of 3.596 g/kg, total nitrogen of 0.226 g/kg, total phosphorus of 0.196 g/kg, and total potassium of 30.328 g/kg. We used sandy soil because it was easy to clean the roots and protected them from damage. Based on the average nitrogen content of northeastern soils (Zhang et al., 2017), we added an additional 10g slow released blended fertilizer (N:P_2_O_5_:K_2_O = 25:12:18) to each pot. Plants of uniform growth were selected in the field and transplanted to the experimental pots at the end of April. Infrared radiation heaters were used to heat the pots which were raised 2°C above throughout the day. In the simulation experiment of nitrogen deposition, wet deposition was used, and complex nitrogen was added at a level of 5 g N m^−2^ year^−1^ in the ratio of ammonium nitrogen:nitrate nitrogen:amide nitrogen = 1:1:1, in four additions, each 1 week apart (Ren et al., 2021).

### Collection and extraction of root exudates

At the seedling stage (marked as the control (SC), warming treatment (SW), and warming with nitrogen addition treatment (SWN), respectively) and the maturity stage (marked as the control (MC), warming treatment (MW), and warming and nitrogen addition treatment (MWN), respectively), 15 plants of the same growth were taken from each treatment, dug up, washed the roots, rinsed with distilled water, placed in culture bottles filled with distilled water, sealed, and grown in the original pots for 24 h (5 plants for each replications, three replicates).

The samples should be mixed with a vortex for 10 s after being thawed from the refrigerator at − 80 °C. Then, take 3 mL of the sample, and put it in a centrifuge tube with 5 mL of liquid nitrogen. After the sample had been totally frozen, place it in the lyophilizer for freeze-drying. Once the samples had been entirely lyophilized, 100µL of a 70% methanol internal standard extract was added. Scroll for 3 min; centrifuge at 12,000 rpm for 10 min at 4 °C. A microporous filter membrane (0.22 µm) was used to filter the supernatant, which was then kept in a sample flask for an LC–MS/MS test.

### UPLC conditions and ESI-Q TRAP-MS/MS analysis

Analysis of sample extracts used a UPLC-ESI-MS/MS system (UPLC, SHIMADZU Nexera X2, https://www.shimadzu.com.cn/; MS, Applied Biosystems 4500 Q TRAP, https://www.thermofisher.cn/cn/en/home/brands/applied-biosystems.html) (UPLC: Column, Agilent SB-C18 (1.8 µm, 2.1 mm × 100 mm). The mobile phase consisted of pure water containing 0.1% formic acid (solvent A) and acetonitrile (solvent B). Sample measurements were analyzed using a gradient program with starting conditions of 95% A and 5% B. A linear gradient injection was programmed from starting conditions of 5% A and 95% B in 9 min. The composition consisted of 5% A and 95% B infused continuously for 1 min. Following that, the 95% A and 5.0% B composition was modified over 1.1 min and maintained for 2.9 min. The flow rate was 0.35 mL per minute. The column oven temperature was set to 40°C. The injection volume was 4 μL. The effluent was bound to an ESI-triple quadrupole-linear ion trap (QTRAP)-MS alternatively (Chen et al., 2013).

Linear ion trap (LIT) and triple quadrupole (QQQ) scans were acquired on a triple quadrupole-linear ion trap mass spectrometer (Q TRAP), AB4500 Q TRAP UPLC/MS/MS System, equipped with an ESI Turbo Ion-Spray interface, running in positive and negative ion mode, and controlled by Analyst 1.6.3 software (AB Sciex). The following were the ESI source operation parameters: ion source, turbo spray; source temperature 550°C; ion spray voltage (IS) 5500 V (positive ion mode)/−4500 V (negative ion mode); the parameters for the ion spray voltage (IS) were 345, 414, and 172 kPa for the ion source gas I (GSI), gas II (GSII), and curtain gas (CUR), respectively; and collision-activated dissociation (CAD), high. Instrument tuning and mass calibration were performed with 10 and 100 μmol/L polypropylene glycol solutions in QQQ and LIT modes, respectively. QQQ scans were acquired as MRM experiments with collision gas (nitrogen) set to medium. Individual MRM transitions were subjected to declustering potential (DP) and collision energy (CE) optimization. A specific set of MRM transitions was monitored for each period according to the metabolites eluted within this period (Chen et al., 2013).

### Root weight measurement

Thirty pots were selected at the conclusion of the experiment to determine root weight. To get dry weight, the root was dehydrated in an oven at 70 °C for 24 h.

### Data analysis

One-way ANOVA with Tukey’s test was used to evaluate the root weight differences among the three treatments.

Root exudate data acquisition and processing were performed as previously described (Chen et al., 2013). Qualitative analysis of MS/MS data was based on self-built database MWDB (Wuhan Meiwei Biotechnology Co., Ltd.) and public database of metabolite information. Using the statistics function prcomp in R (www.r-project.org), unsupervised principle component analysis (PCA) was performed. Prior to unsupervised PCA, the data were unit variance scaled. Model stability reliability was predicted according to orthogonal partial least squares discriminant analysis (OPLS-DA) method. The data was log transform (log_2_) and mean centering before OPLS-DA. Variable importance in project (VIP) was extracted from the OPLS-DA result, which comprised score plots and permutation plots, using the R package MetaboAnalystR. To avoid overfitting, a permutation test with 200 permutations was performed. Differentiated metabolites were screened by multidimensional statistical VIP values and fold changes. For correlation pathway analysis, the related difference metabolites were uploaded to the KEGG database (http://www.kegg.jp/kegg/pathway.html). Pathways with significantly regulated metabolites mapped to were then fed into metabolite sets enrichment analysis (MSEA), and their significance was determined by the *p* value in a hypergeometric test.

## Results

### Root weight responses to environmental changes

**Fig. 1:**
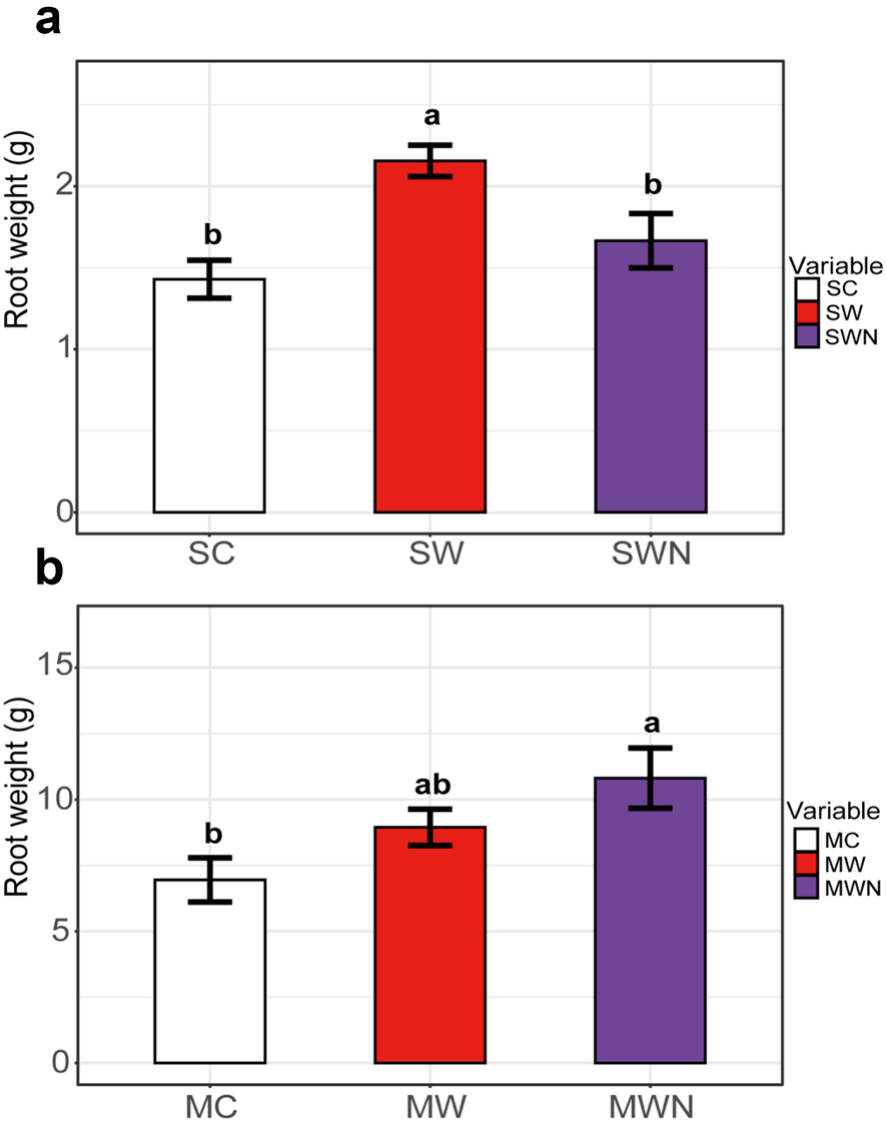
Root weight in different periods. **a** seedling stage; **b** maturity stage

The root weight under the control group (SC) was significantly lower than that in SW while there was no significant difference with that in SWN at the seedling stage (Fig. 1a); conversely, the root weight under the control group (MC) was significantly lower than that in MWN, while there was no significant difference with that in MW at the maturity stage (Fig. 1b). This showed that warming promoted plant growth, and nitrogen addition reduced the advantage of rapid growth brought by temperature increase at seedling stage but promoted plant growth at maturity stage.

### Overview of the root exudates

**Fig. 2:**
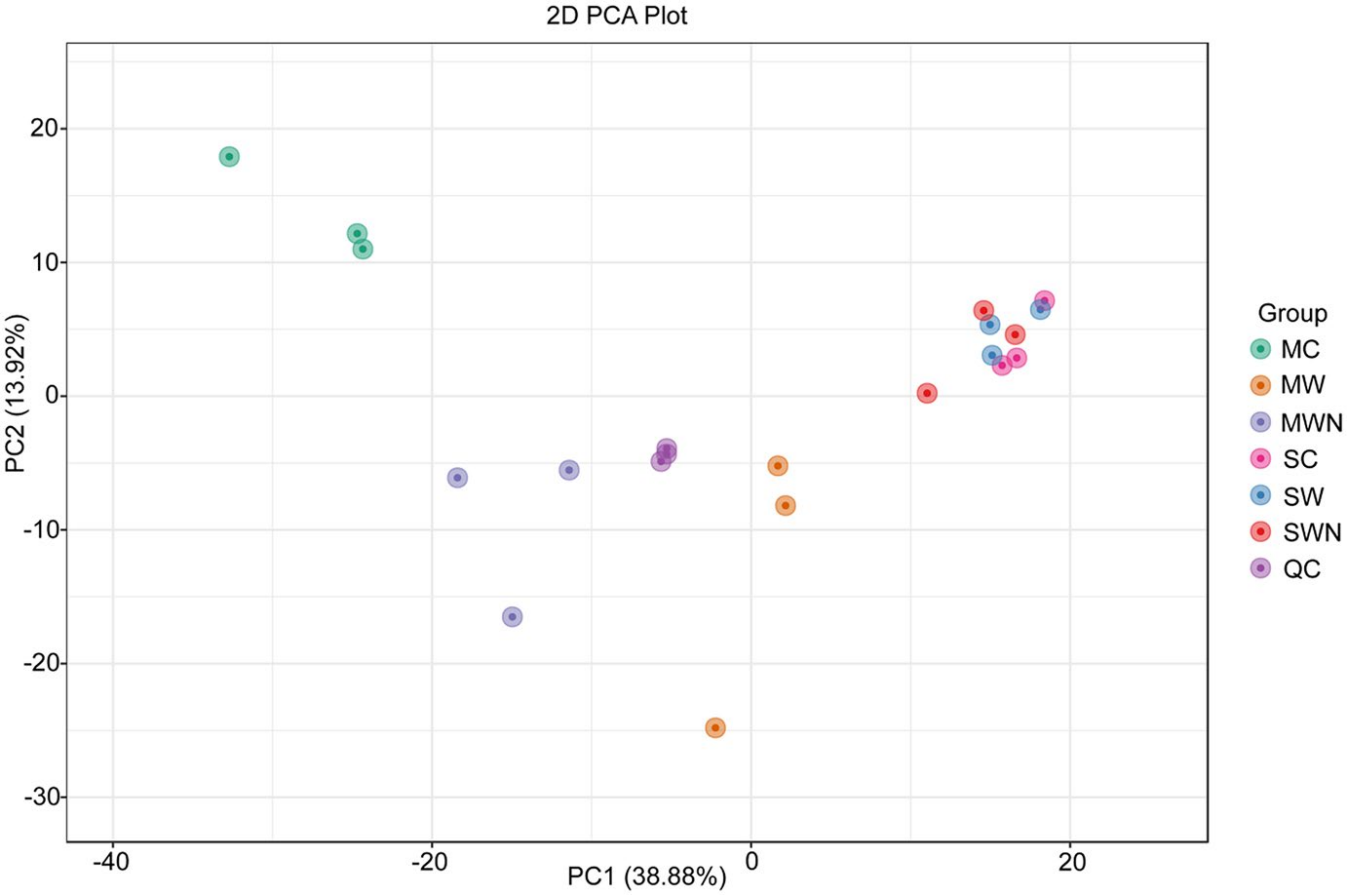
PCA analysis of seven groups of samples

To gain a more clearly picture of the metabolite changes in different treatments, root exudates in the samples were identified by widely targeted metabolomic techniques on the UPLC-MS platform. A total of 684 metabolites were detected, including 61 amino acids and derivatives, 114 nucleotides and derivatives, 178 organic acids, 138 lipids, 296 phenolic acids, 49 flavonoids, 40 lignans and coumarins, 49 alkaloids, 17 terpenoids, and 93 others (Table S1). A quality control (QC) sample was included into every ten test samples to ensure the validity of the analytical method. Sample extracts were combined to make QC samples. QC samples were grouped together, suggesting the entire analysis was stable and repeatable (Fig. 2). The results indicated that the first and second principal components together explained 52.8% of the variation between samples, with a clear pattern on different treatments.

### Identification of differential root exudates

**Fig. 3:**
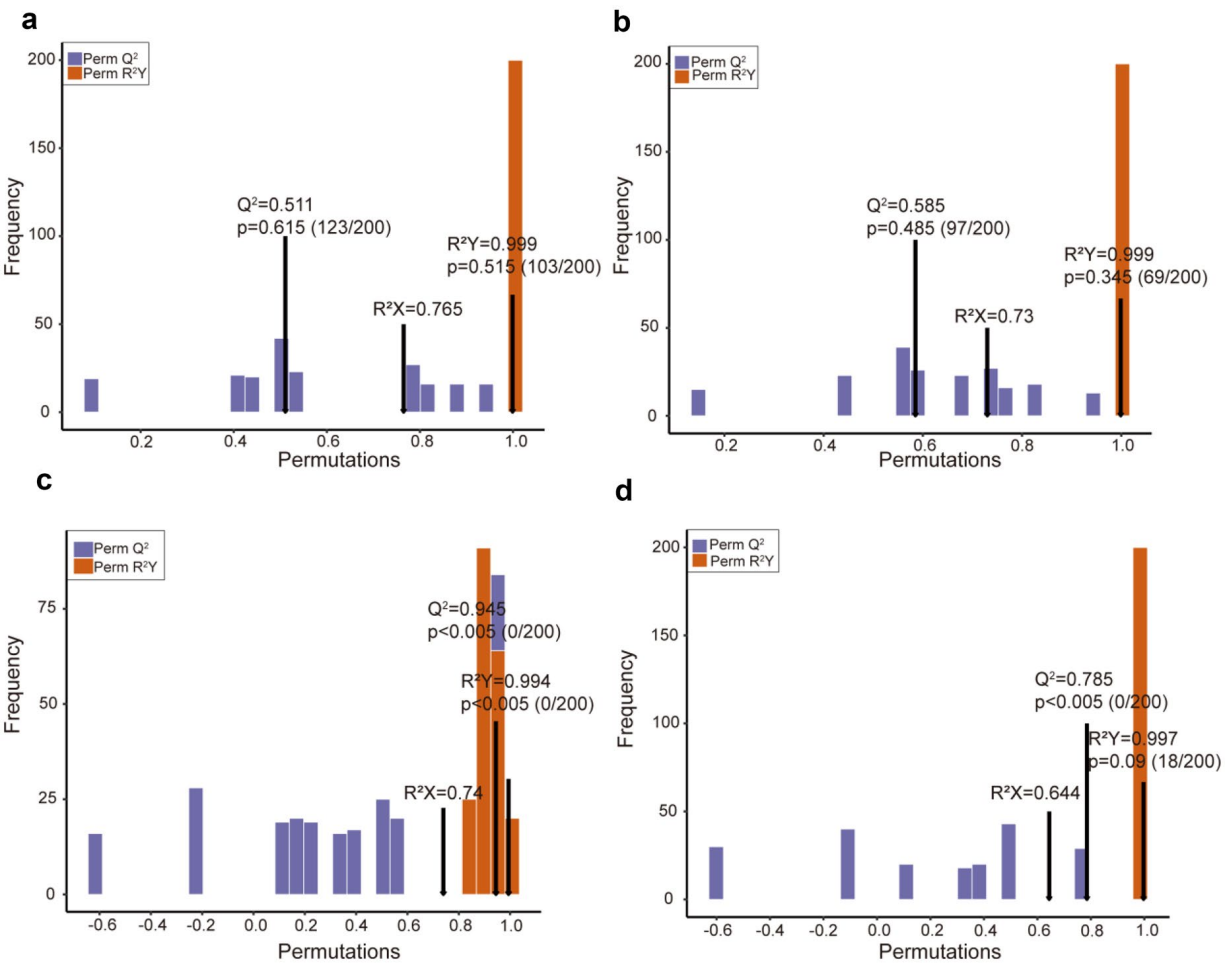
Plot of OPLS-DA scores for each group. **a** SC vs SW; **b** SW vs SWN; **c** MC vs MW; **d** MW vs MWN

The score plot was obtained by pairwise comparisons with OPLS-DA model (Fig. 3). *Q*^2^ represented prediction ability, whereas *R*^2^ *X* and *R*^2^ *Y* indicated the rates of interpretation of the *X* and *Y* matrices, respectively. The results of all two-by-two comparisons demonstrated that the model was suitable because the scores of *R*^2^ *Y* and *Q*^2^ were higher than 0.5.

**Fig. 4:**
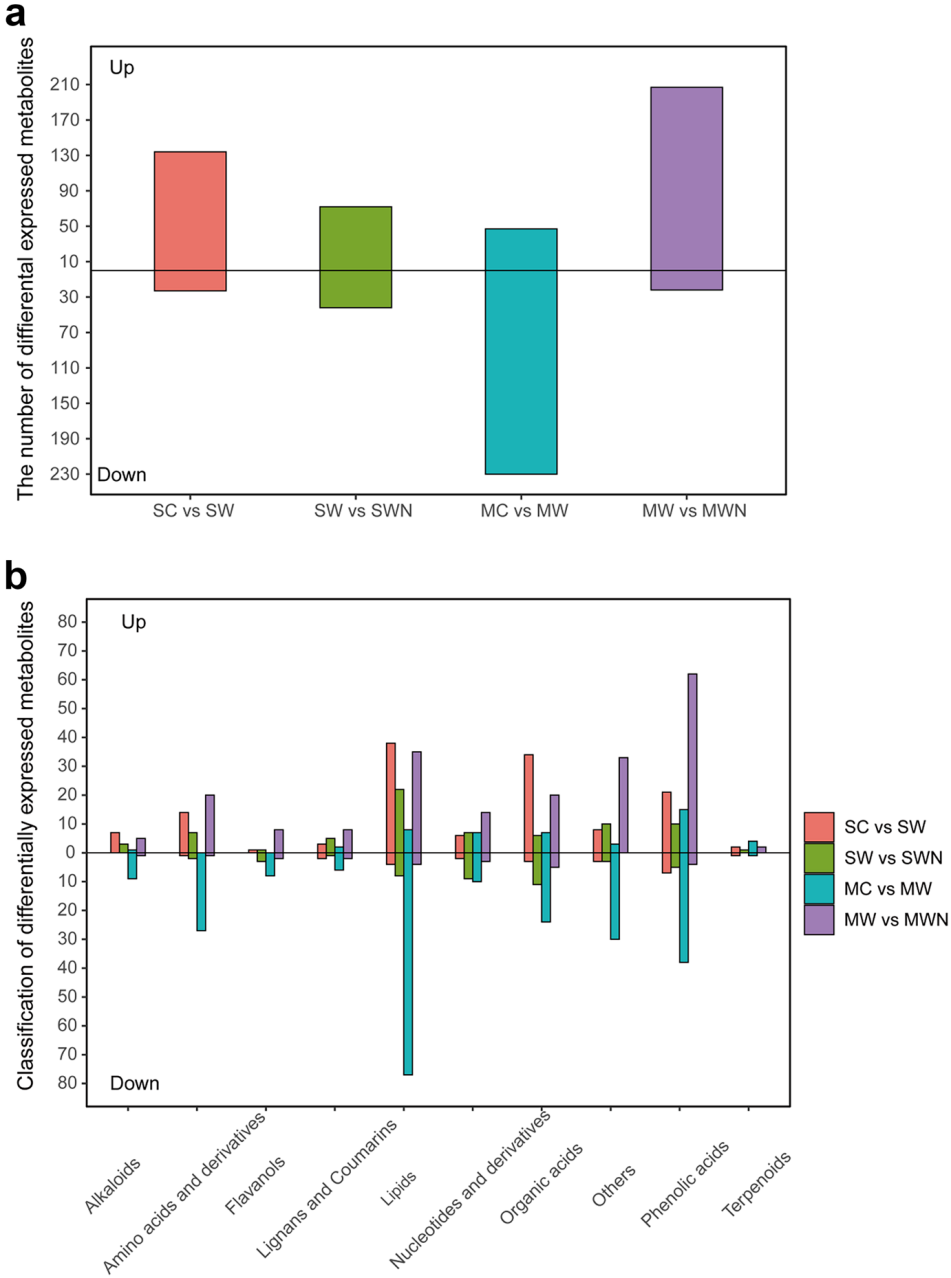
Quantity comparison of differentially expressed metabolites in each pair of root exudates of *A. trifida* (**a**) and classification of differentially expressed metabolites in four pairs of comparisons (**b**)

We had chosen significant differential root exudates with a fold change of ≥ 2, a fold change of ≤ 0.5, and a VIP ≥ 1. As shown in Fig. 4a, the differential metabolite composition of the different treatments at different periods, where the number of upregulated compounds was higher than the number of downregulated compounds, suggested that the key physiological and metabolic activities may be activated by warming and increased nitrogen deposition at different periods. The diverse metabolites generated across the various treatments were further categorized and contrasted. These metabolites that showed differential expression were divided into 10 groups, mainly focusing on amino acids, phenolic acids, organic acids, and lipids (Table S2). In SW, 14 amino acids, 38 lipids, 34 organic acids, and 21 phenolic acids were upregulated, and 1 amino acid, 4 lipids, 3 organic acids, and 7 phenolic acids were downregulated compared with those in SC; 7 amino acids, 22 lipids, 6 organic acids, and 10 phenolic acids were upregulated, and 2 amino acids, 8 lipids, 11 organic acids, 5 phenolic acids were downregulated in SWN compared with those in SW. In MW, 8 lipids, 7 organic acids, and 15 phenolic acids were upregulated, and 27 amino acids, 77 lipids, 24 organic acids, and 38 phenolic acids were downregulated compared with those in MC; 20 amino acids, 39 lipids, 25 organic acids, and 66 phenolic acids were upregulated, and 1 amino acid, 4 lipids, 5 organic acids, and 4 phenolic acids were downregulated in MWN compared with those in MW (Fig. 4b).

It could be inferred that under warming condition lipids, phenolic acids and organic acids were much higher upregulated than other metabolites at seedling stage. Nitrogen deposition did not increase the secretion of root exudates of *A. trifida* at seedling stage, but at mature stage was the opposite, especially for phenolic acid compounds. Among them, phenolic acids were significantly upregulated in all four treatments, with phenolics such as p-coumaric acid, 3-(4-hydroxyphenyl)-1-propanol, and 4-methylphenol at seedling stage and 6-O-glucosyl-caffeoylbenzoic acid and 2-methylbenzoic acid at maturity. Phenolic acids had allelopathy such as inhibition of seed germination (Jian et al., 2010) and alteration of soil microbial load and activity (Sparling et al., 1981), and phenolic acids may act as direct or indirect means for invasive plants to engage in competition (Callaway & Aschehoug, 2000; Ridenour & Callaway, 2001), providing a competitive advantage. It can be deduced that warming and nitrogen deposition increased phenolic acids secretion in *A. trifida* at different growth stages, giving it a stronger allelopathy.

### KEGG annotation and enrichment analysis of differential metabolites

**Fig. 5:**
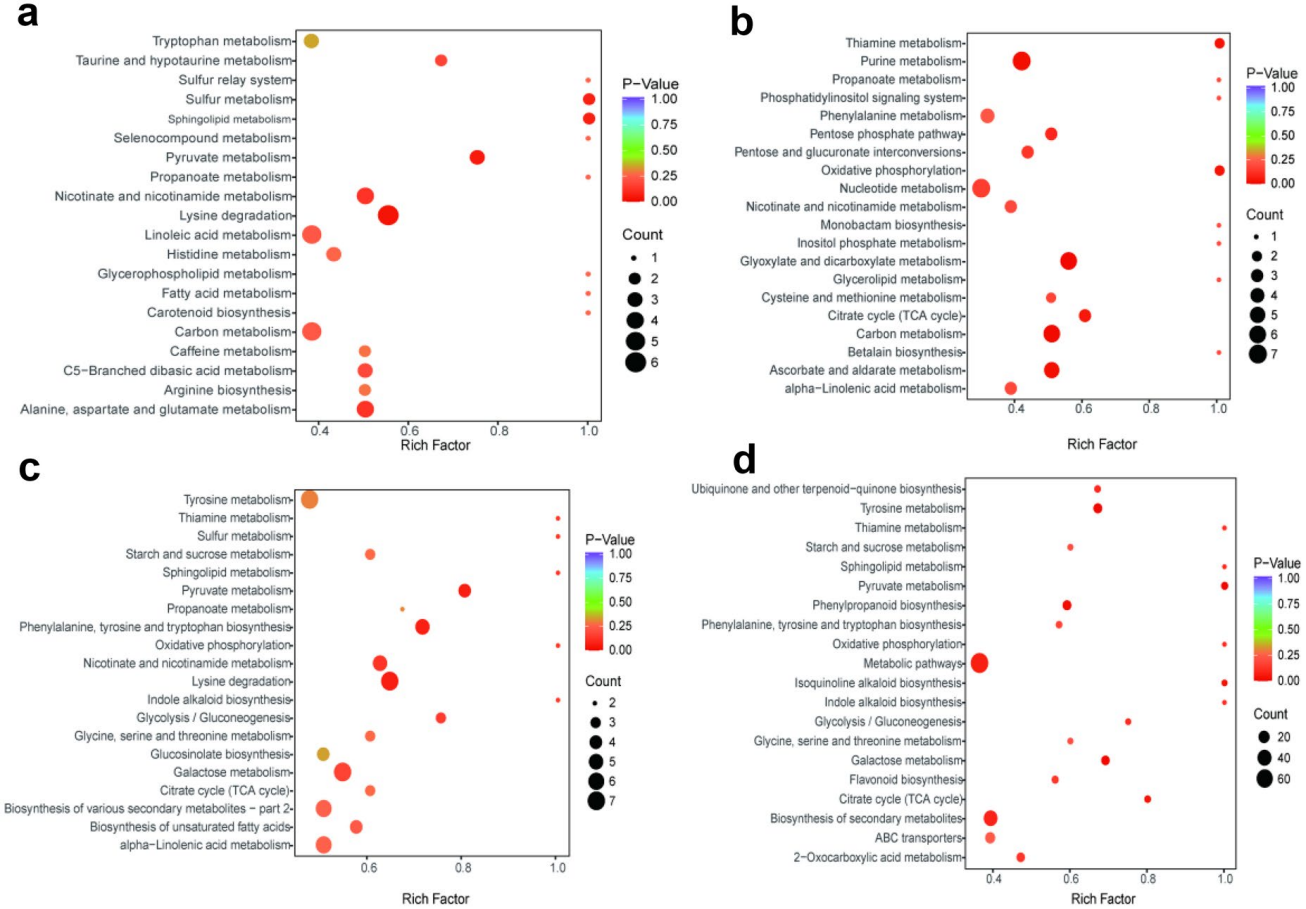
KEGG annotations for pairwise comparison of root exudates at different periods. **a** SC vs SW; **b** SW vs SWN; **c** MC vs MW; **d** MW vs MWN

In order to further understand the pathways involved in the metabolites of different comparison groups, the different metabolites were linked to the KEGG database. The pathways with high enrichment of different metabolites were found using enrichment analysis on the annotated results. In Fig. 5, we had shown the enrichment factor using the ratios of the number of different metabolites detected to the total number of metabolites. A higher ratio represents a higher enrichment. As *p* value tends to be 0, the enrichment became more significant. The size of the bubble represents the number of metabolites enriched in the corresponding pathway. The results in Fig. 5 showed that the metabolite differences in SW were mainly annotated on lysine metabolism; in SWN, they were annotated on glyoxylate and dicarboxylate metabolism, carbon metabolism, and purine metabolism; and in MWN they were annotated on tyrosine metabolism and phenylpropanoid biosynthesis.

## Discussion

Climate warming can directly affect the photosynthetic rates, change phenology, prolong growth season, and then affect the root biomass; secondly, temperature can affect the decomposition rate of soil organic matter and the availability of nutrients, resulting in changes in root biomass (Li et al., 2020). A meta-analysis of 964 paired observations from 177 publications showed that warming promoted root biomass in terrestrial ecosystems (Wang et al., 2021). Nitrogen deposition increased root biomass in MWN but no significant increase in SWN, which may be due to the increased of available nitrogen content in the soil, so that *A. trifida* reduced the input of belowground parts in seedling stage (Ren et al., 2019). Larger roots can buffer the impact of abiotic stress and may further expand the invasion scope by increasing the belowground competitiveness of invasive plants. Under the combined impact of nitrogen deposition and climate warming, *A. trifida* may have stronger invasiveness.

Root exudates can continuously adjust their composition and quantity in response to changes in the surrounding environment, and warming promotes an increase in root exudates in most plants (Uselman et al., 2000; Yin et al., 2013) while changing the chemical composition of root exudates (Qiao et al., 2014; Xiong et al., 2020). The content of some organic acids, amino acids, lipids, phenolic acids, and other metabolites in the root exudates of *A. trifida* changed significantly under both warming only and warming with nitrogen deposition, indicating that *A. trifida* can adapt to climate change by regulating its metabolites.

Low-molecular-weight organic acids have certain acid effects and carboxyl complexation in the soil environment. They have important roles in altering soil physicochemical properties, mineral solubilization, inter-root nutrition, and alleviation of heavy metal toxicity (Lundström & Öhman, 1990; Wu et al., 2003; Bais et al., 2006). A significant upregulation of organic acids such as abscisic acid, pipecolic acid, α-ketoglutaric acid, and succinic acid was observed in SW. ABA in the root system leads to enhanced biosynthesis of osmoregulatory substances, such as proline (Sardans et al., 2014). Increased abscisic acid in *A. trifida* induced and suggested a strong defense capacity. Warming mainly promoted the accumulation of organic acids such as succinic acid involved in TCA cycle for respiration metabolism. And the downstream product of lysine catabolism, pipecolate, has been shown to have antiradical effects such as osmoregulation and resistance to pathogenic bacterial infection (Bernsdorff et al., 2016; Kiyota et al., 2015). We also observed no significant changes in organic acid in MWN. The composition and secretion of organic acids in the roots of most plants were significantly altered under low phosphorus conditions. Under phosphorus stress, low-molecular-weight carboxylates (such as malate, citrate, and oxalate) secreted by root can displace immobilized P from P-containing inorganic and organic compounds in soil; phenolics also induced production and secretion into the rhizosphere. These all help to promote phosphorus uptake by plants (Chai & Schachtman, 2022). Long-term N deposition alleviated soil P limitation (Chen et al., 2020), and it is possible that N deposition led to reduced P limitation, so that *A. trifida* reduced the secretion of organic acids.

Amino acids are common low-molecular-weight compounds in root exudates. Warming has significantly promoted amino acids secretion in *A. trifida*. In seedling stage, some amino acids such as cyclic (serine-proline), proline, and L-methionine sulfoxide increased. In maturity stage, there was no difference. When plants are subjected to external adversity stress, large amounts of reactive oxygen species are produced, resulting in the oxidation of methionine into methionine sulfoxide. This will lead to the reduction or loss of protein biological activity and the inability to perform normal functions (Boschi-Muller et al., 2008; Levine et al., 1996). The increased content of methionine sulfoxide indicated oxidative damage in *A. trifida* when temperature increased. Proline is one of the plant proteins widely existing in plants in free state. Under stress conditions such as drought and high temperature, proline accumulates in large amounts in plants and is an important osmoregulatory substance (Verbruggen & Hermans, 2008). In addition, proline plays an important role in the stabilization of biomolecular structures, membranes, and subcellular structures and in the scavenging of reactive oxygen species (Kaur & Asthir, 2015). It was also observed qualitative similarity of amino acids in SWN and WN. And amino acids were reduced in maturity stage, which is probably related to difference of the plant growth stage.

The phenylpropanoid biosynthesis pathway is one of the major secondary metabolic pathways in plants, produces a large number of antioxidants, and shows strong plasticity in the process of plant development and in response to changing environment (Dong & Lin, 2021). In our study, the phenylpropanoid biosynthesis pathway was found to be significantly enhanced in MWN. For example, caffeic acid and ferulic acid are lignin precursors (Vincent et al., 2005). Cell structure is strengthened by lignin, and it has been demonstrated that lignin accumulation is linked to dehydration tolerance (Magalhaes Silva Moura et al., 2010; Xiao et al., 2021). Coumaric acid, ferulic acid, and other phenolic acids also have strong allelopathy. When plants face environmental stresses such as water shortage, fertilizer shortage, and high temperature, they can inhibit the growth of other plants around them by releasing allelochemicals, thus increasing their relative competition for nutrients and water. Chlorogenic acid has possible roles as defense compounds or as potential antioxidants (Petersen et al., 2009). At the same time, some allelochemicals help plants absorb nutrients such as N, P, and metal ions to improve their physiological effects such as resistance to stress, thus increasing their relative competition under adverse conditions, which will have an indirect inhibitory effect on other plants. The latter is more likely to be the main reason for the increase of allelochemicals under environmental stress. It can be seen that *A. trifida* in maturation stage can resist the oxidative damage caused by warming through the phenylpropanoid biosynthesis pathway and produce large amounts of phenolic acids to establish an invasion advantage after nitrogen deposition.

In plants, tyrosine is re-synthesized by the shikimic acid pathway, which also produces phenylalanine and tryptophan (Maeda & Dudareva, 2012). Additionally, tyrosine is the precursor for the vital plant compounds tocopherols, plastoquinone, and ubiquinone (Xu et al., 2020). At the mature stage, *A. trifida* increased the secretion of tyrosine, homogentisate, gentisic acid, homovanillate, and other phenolic acid compounds in root exudates after nitrogen addition. The reversible amino reaction of tyrosine yields 4-hydroxyphenylpyruvic acid, and 4-hydroxyphenylpyruvic acid can be converted to homogentisate by 4-hydroxyphenylpyruvate dioxygenase (Xu et al., 2020). The availability of homogentisate may limit the production of tocochromanol in some plant species and organs (Zhang et al., 2013). In addition, the non-oxidative deamination of tyrosine generates 4-coumaric acid, which is involved in the phenylpropanoid biosynthesis pathway.

At the warming and nitrogen addition treatment, we observed a decrease in organic acids and phenolic acids at the seedling stage and a substantial increase in phenolics at maturity stage than under warming alone. It was possible that nitrogen addition inhibited plant inputs of organic C to the soil, but invasive plants may have strong phenotypic plasticity and more flexible strategies for symbiotic association with soil microbes and obtain resources from the soil (Xu et al., 2021). We hypothesized that N deposition reduced belowground C input in the early stages of plant growth and was used more to maintain aboveground growth, giving invasive plants a dispersal advantage in the later stages of plant growth. At the same time, phenolic acids have soil legacy effects that may be associated with more stable changes in nutrient cycling and microbial communities, which can harm native plants (Cipollini et al., 2012). In conclusion, root exudates of plants were more invasive after nitrogen deposition. Nitrogen deposition met the nutrient requirements of the overgrowth in the early growth stage due to warming. The high number of phenolic compounds produced after nitrogen deposition may continue to affect native plant growth.

The traditional view is that high temperature is an important factor limiting the growth of *A. trifida*, so it grows well in temperate region with low temperatures, and its main distribution is also in the northern temperate zone (Essl et al., 2015). It does not grow well in southern China where the temperature is higher. *A. trifida* has been found in the Sichuan and Guizhou provinces of China, and there has been an outbreak trend observed. This suggests that this particular population of *A. trifida* has adapted to the local climate and has undergone rapid evolution in temperature adaptation. Sichuan Province, located in southwestern China, spans several geomorphic units including the Tibetan Plateau, the Hengduan Mountains, the Qinba Mountains, and the Sichuan Basin, and is one of the global biodiversity hotspots and one of the precious species gene pools in both China and worldwide. However, the Sichuan natural ecosystem is generally fragile and natural disasters are frequent. Once *A. trifida* invades natural ecosystem, the impact of its destruction is mostly irreversible and often leads to species extinction. Habitat restoration is very difficult and more costly. Therefore, concentrated eradication measures should be taken in the areas of *A. trifida* outbreak to prevent further damage.

## Conclusion

Widely targeted metabolomics was used to identify 684 metabolites in the root exudates of *A. trifida*. The differential metabolites were mainly lipids, phenolics, and organic acids. Increased temperature promoted the secretion of metabolites such as cyclic (serine-proline), L-methionine sulfoxide, abscisic acid, caffeic acid, and ferulic acid. Moreover, nitrogen deposition increases the secretion of phenolic acid compounds. KEGG analysis of the metabolic pathways revealed that tyrosine metabolism and phenylpropanoid biosynthesis may have a major role in osmoregulation and allelopathy after nitrogen deposition. In conclusion, nitrogen deposition further increased the invasion risk of *A. trifida*.

## Supplementary Information

Below is the link to the electronic supplementary material.

Supplementary file1 (DOCX 40 KB)

Supplementary file2 (DOCX 99 KB)

## Data Availability

The datasets generated during and/or analyzed during the current study are available from the corresponding author upon reasonable request.
