# Supplementary material for: Nitrogen deposition further increases Ambrosia trifida root exudate invasiveness under global warming
Source: Environ Monit Assess. 2023 May 30;195(6):759. doi: 10.1007/s10661-023-11380-w (PMC10229694; doi:10.1007/s10661-023-11380-w)
Supplement: Supplementary file 1 — Supplementary file1 (DOCX 40 KB) [file 10661_2023_11380_MOESM1_ESM.docx]

**Table S1:** Name, clsss and formula of 685 detected compounds

| Class | Compounds | Formula |
| --- | --- | --- |
| Alkaloids | Putrescine  Choline  4-Aminophenol  N-Benzylmethylene isomethylamine  Phenethylamine  1-Methylhistamine  Imidazole-4-acetate  Methyl nicotinate  6-Hydroxynicotinic acid  Histidinol  N-Acetylcadaverine  Spermidine  4-Hydroxymandelonitrile  Vanillylamine  DL-2-Aminoadipic acid  Quinolinic acid  O-Phosphocholine  N-(4-Aminobutyl)benzamide  Spermine  2-(Acetylamino)-3-phenyl-2-propenoic acid  Lumichrome  Hexadecyl ethanolamine  N-Feruloyltyramine  N-Oleoylethanolamine  Cocamidopropyl betaine  p-Coumaroylferuloylcadaverine  N'-p-Coumaroylagmatine-glucoside  Diferuloylcadaverine | C4H12N2  C5H13NO  C6H7NO  C8H9N  C8H11N  C6H11N3  C5H6N2O2  C7H7NO2  C6H5NO3  C6H11N3O  C7H16N2O  C7H19N3  C8H7NO2  C8H11NO2  C6H11NO4  C7H5NO4  C5H15NO4P+  C11H16N2O  C10H26N4  C11H11NO3  C12H10N4O2  C18H39NO  C18H19NO4  C20H39NO2  C19H38N2O3  C24H28N2O5  C20H30O7N4  C25H30N2O6 |
| Amino acids and derivatives | L-Alanine | C3H7NO2 |
|  | N,N-Dimethylglycine  L-Proline  L-Valine  Cycloleucine  Trans-4-Hydroxy-L-proline  L-Isoleucine*  L-Leucine*  L-Allo-isoleucine*  L-Norleucine*  L-Asparagine  L-Cyclopentylglycine  D-Proline betaine  L-Glutamine  L-Lysine  L-Glutamic acid  L-Methionine  L-Methionine Sulfoxide  L-Phenylalanine  Cyclo(L-Ala-L-Pro)  1-Methylhistidine  N-Acetyl-L-leucine  L-Tyrosine  Cyclo(Ser-Pro)  N-Acetyl-L-Glutamine  N6-Acetyl-L-lysine  L-Glycyl-L-isoleucine*  N-Glycyl-L-leucine*  Arginine methyl ester  N-Monomethyl-L-arginine  Trimethyllysine  L-Homocitrulline  Cyclo(Pro-Pro)  L-Tyrosine methyl ester  Cyclo(Pro-Val)  L-2-chlorophenylalanine  L-Tryptophan  cinnamoylglycine  N-Acetyl-L-phenylalanine  N-Acetyl-DL-phenylalanine  Cyclo(Pro-Leu)  3-(2-Naphthyl)-L-alanine  N-Acetyl-L-Arginine  Glycylphenylalanine  N-Acetyl-L-tyrosine  L-Prolyl-L-Leucine  L-Valyl-L-Leucine  N'-Formylkynurenine  L-Alanyl-L-Phenylalanine  Cyclo(D-Phe-L-Pro)  Cyclo(Pro-Phe)  L-Leucyl-L-Leucine  N-(3-Indolylacetyl)-L-alanine  N-Acetyl-L-Tryptophan  L-Isoleucyl-L-Aspartate  L-γ-Glutamyl-L-leucine  Phenylacetyl-L-glutamine  L-Valyl-L-Phenylalanine  L-Leucyl-L-phenylalanine  L-Aspartyl-L-Phenylalanine  L-Phenylalanyl-L-phenylalanine | C4H9NO2  C5H9NO2  C5H11NO2  C6H11NO2  C5H9NO3  C6H13NO2  C6H13NO2  C6H13NO2  C6H13NO2  C4H8N2O3  C7H13NO2  C7H14NO2+  C5H10N2O3  C6H14N2O2  C5H9NO4  C5H11NO2S  C5H11NO3S  C9H11NO2  C8H12N2O2  C7H11N3O2  C8H15NO3  C9H11NO3  C8H12N2O3  C7H12N2O4  C8H16N2O3  C8H16N2O3  C8H16N2O3  C7H16N4O2  C7H16N4O2  C9H20N2O2  C7H15N3O3  C10H14N2O2  C10H13NO3  C10H16N2O2  C9H10ClNO2  C11H12N2O2  C11H11NO3  C11H13NO3  C11H13NO3  C11H18N2O2  C13H13NO2  C8H16N4O3  C11H14N2O3  C11H13NO4  C11H20N2O3  C11H22N2O3  C11H12N2O4  C12H16N2O3  C14H16N2O2  C14H16N2O2  C12H24N2O3  C13H14N2O3  C13H14N2O3  C10H18N2O5  C11H20N2O5  C13H16N2O4  C14H20N2O3  C15H22N2O3  C13H16N2O5  C18H20N2O3 |
| Flavonoids | Pinocembrin (Dihydrochrysin)  5-Hydroxy-7-methoxyflavone  Baicalein  Norwogonin  Pinostrobin  Naringenin (5,7,4'-Trihydroxyflavanone)  Afzelechin (3,5,7,4'-Tetrahydroxyflavan)  Eriodictyol (5,7,3',4'-Tetrahydroxyflavanone)  Chrysoeriol  Rhamnocitrin (7-Methylkaempferol)  Quercetin  Myricetin  Ayanin (3',5-Dihydroxy-3,4',7-Trimethoxyflavone)  Tangeretin (4',5,6,7,8-Pentamethoxyflavone)  Quercetin-3-O-Sulfonate  Nobiletin (5,6,7,8,3',4'-Hexamethoxyflavone)  3',5,5',7-Tetrahydroxyflavanone-7-O-glucoside  Quercetin-3-O-glucoside (Isoquercitrin)  Apigenin-7-O-neohesperidoside (Rhoifolin)  Apigenin-6,8-di-C-glucoside (Vicenin-2)  Luteolin-7-O-neohesperidoside (lonicerin)  Diosmetin-7-O-rutinoside (Diosmin)  Quercetin-3-O-(4''-O-glucosyl)rhamnoside | C15H12O4  C16H12O4  C15H10O5  C15H10O5  C16H14O4  C15H12O5  C15H14O5  C15H12O6  C16H12O6  C16H12O6  C15H10O7  C15H10O8  C18H16O7  C20H20O7  C15H10O9S  C21H22O8  C21H22O11  C21H20O12  C27H30O14  C27H30O15  C27H30O15  C28H32O15  C27H30O16 |
| Lignans and Coumarins | 6-MethylCoumarin  4-Hydroxycoumarin  Daphnetin  Angelicin  Ayapin  Coumarin-3-carboxylic Acid  Isoscopoletin (6-Hydroxy-7-Methoxycoumarin)  scopoletin (7-Hydroxy-5-methoxycoumarin)  6,7-Dihydroxy-4-methylcoumarin  Bergaptol  Xanthotoxol  Scoparone (6,7-Dimethoxycoumarin)  Fraxetin (7,8-Dihydroxy-6-methoxycoumarin)  6,7-Dimethoxy-4-methylcoumarin  Fraxidin (8-Hydroxy-6,7-dimethoxycoumarin)*  isofraxidin*  O-Feruloyl 3-hydroxycoumarin  Daphnin  Esculetin-7-O-glucoside  Esculetin-7-O-quinic acid  scopoletin-7-O-glucoside (Scopolin)  Matairesinol  Pinoresinol*  Epipinoresinol*  Isolariciresinol  (7R,8S)-Dihydrodehydrodiconiferyl alcohol  scopoletin-7-O-glucuronide  Arctigenin  Isofraxidin-7-O-glucoside  Trachelogenin  syringaresinol  Dihydrodehydrodiconiferyl alcohol-4-O-glucoside  lariciresinol-4'-O-glucoside  Olivil-4'-O-glucoside  5'-Methoxymatairesinoside  Eucommin A  Lappaol C  syringaresinol-4'-O-glucopyranosid  Medioresinol-4'-O-(6'''-acetyl)glucoside  syringaresinol-4'-O-(6''-acetyl)glucoside | C10H8O2  C9H6O3  C9H6O4  C11H6O3  C11H8O3  C10H6O4  C10H8O4  C10H8O4  C10H8O4  C11H6O4  C11H6O4  C11H10O4  C10H8O5  C12H12O4  C11H10O5  C11H10O5  C19H14O6  C15H16O9  C15H16O9  C16H16O9  C16H18O9  C20H22O6  C20H22O6  C20H22O6  C20H24O6  C20H24O6  C16H16O10  C21H24O6  C17H20O10  C21H24O7  C22H26O8  C26H34O11  C26H34O11  C26H34O12  C27H34O12  C27H34O12  C30H34O10  C28H36O13  C29H36O13  C30H38O14 |
| Lipids | Octanoic acid  Undecylic Acid  10-Hydroxydecanoic acid  Dodecanoic acid (Lauric acid)  δ-Tridecalactone  Tridecanoic Acid  Undecanedioic acid  Myristoleic acid  2-Dodecenedioic acid  Myristic Acid  Dodecanedioic aicd  Palmitaldehyde  Pentadecanoic Acid  12-Methyltetradecanoic Acid  Tridecanedioic acid  1-O-Caffeoylglycerol  Palmitoleic Acid  Tetradecanedioic acid  10-Heptadecenoic Acid  16-Hydroxyhexadecanoic acid  Hexadecylsphingosine  9,12-Octadecadien-6-Ynoic Acid  α-Linolenic Acid*  Punicic acid (9Z,11E,13Z-octadecatrienoic acid)  γ-Linolenic Acid*  Octadeca-11E,13E,15Z-trienoic acid  Linoleic acid  Oleamide (9-Octadecenamide)  Oleic acid  Elaidic Acid  Hexadecanedioic acid  9,16-Dihydroxypalmitic acid  13-KODE; (9Z,11E)-13-Oxooctadeca-9,11-dienoic acid  2R-hydroxy-9Z,12Z,15Z-octadecatrienoic acid  17-Hydroxylinolenic acid  13S-Hydroxy-9Z,11E,15Z-octadecatrienoic acid  9-Oxo-12Z-Octadecenoic acid  15(R)-Hydroxylinoleic Acid  9S-Hydroxy-10E,12Z-octadecadienoic acid  9(10)-EpOME;(9R,10S)-(12Z)-9,10-Epoxyoctadecenoic acid  13(S)-HODE;13(S)-Hydroxyoctadeca-9Z,11E-dienoic acid  1-Eicosanol  3-Dehydrosphinganine  12-HYDROXYOCTADECANOIC ACID  3-Hydroxyoctadecanoic Acid  1-Monomyristin  Dihomo-gamma-linolenic acid; (8Z,11Z,14Z)-Icosatrienoic acid  Eicosadienoic acid  9-Hydroperoxy-10E,12,15Z-octadecatrienoic acid  13S-Hydroperoxy-6Z,9Z,11E-octadecatrienoic acid  9-Hydroxy-12-oxo-10(E),15(Z)-octadecadienoic acid  13(s)-hydroperoxy-(9z,11e,15z)-octadecatrienoic acid  13-Hydroxy-9Z,11E-octadecadienoic acid  Eicosenoic acid  7S,8S-DiHODE; (9Z,12Z)-(7S,8S)-Dihydroxyoctadeca-9,12-dienoic acid  13S-Hydroperoxy-9Z,11E-octadecadienoic acid  9-Hydroxy-12-oxo-15(Z)-octadecenoic acid  9-Hydroperoxy-9Z,11E-Octadecadienoic Acid  12,13-DHOME; (9Z)-12,13-Dihydroxyoctadec-9-enoic acid  Hydroxy ricinoleic acid  4-Hydroxysphinganine  12-Oxo-5,8,10,14-eicosatetraenoic acid  9,12,13-Trihydroxy-10,15-octadecadienoic acid  9,10,13-Trihydroxy-11-Octadecenoic Acid  9,10-Dihydroxy-12,13-epoxyoctadecanoic acid  15-Hydroperoxyicosatetraenoic acid  5,6,15-Trihydroxy-7,9,11,13-eicosatetraenoic acid  2-α-Linolenoyl-glycerol*  1-α-Linolenoyl-glycerol*  2-Linoleoylglycerol*  1-Oleoyl-Sn-Glycerol  1,3-O-Dicaffeoylglycerol  LysoPE 14:0*  LysoPE 14:0(2n isomer)*  LysoPE 15:1*  LysoPE 15:1(2n isomer)*  LysoPE 15:0(2n isomer)*  LysoPE 15:0*  LysoPE 16:3  LysoPE 16:1*  LysoPE 16:1(2n isomer)*  LysoPE 16:0(2n isomer)*  LysoPE 16:0*  LysoPE 17:1(2n isomer)*  LysoPE 17:1*  LysoPC 14:0  LysoPE 18:3(2n isomer)*  LysoPE 18:3*  LysoPE 18:2(2n isomer)*  LysoPE 18:2*  LysoPE 18:1(2n isomer)*  LysoPE 18:1*  LysoPC 15:0*  LysoPE 18:0(2n isomer)*  LysoPC 15:0(2n isomer)*  LysoPE 18:0*  LysoPG 16:0  LysoPC 16:4  LysoPC 16:2(2n isomer)*  LysoPC 16:2*  LysoPC 16:1(2n isomer)*  LysoPC 16:1*  LysoPC 16:0(2n isomer)*  LysoPC 16:0*  LysoPE 20:5  LysoPE 20:4(2n isomer)*  LysoPE 20:4*  LysoPE 20:3(2n isomer)*  LysoPE 20:3*  LysoPC 17:2  LysoPE 20:2*  LysoPE 20:2(2n isomer)*  LysoPC 17:1  LysoPC 17:0*  LysoPC 17:0(2n isomer)*  LysoPC 18:4  LysoPC 18:3(2n isomer)*  LysoPC 18:3*  LysoPC 18:2*  LysoPC 18:2(2n isomer)*  LysoPC 18:1(2n isomer)*  LysoPC 18:1*  LysoPC 18:0(2n isomer)*  LysoPC 19:1  LysoPC 19:0  LysoPC 20:5  LysoPC 20:4  LysoPC 20:3  LysoPC 20:2(2n isomer)*  LysoPC 20:2*  LysoPC 22:6  LysoPC 22:5*  LysoPC 22:5(2n isomer)*  LysoPC 22:4  1-Linolenoyl-rac-glycerol-diglucoside  1-Linoleoylglycerol-2,3-di-O-glucoside*  1-Linoleoyl-sn-glycerol-diglucoside  2-Linoleoylglycerol-1,3-di-O-glucoside* | C8H16O2  C11H22O2  C10H20O3  C12H24O2  C13H24O2  C13H26O2  C11H20O4  C14H26O2  C12H20O4  C14H28O2  C12H22O4  C16H32O  C15H30O2  C15H30O2  C13H24O4  C12H14O6  C16H30O2  C14H26O4  C17H32O2  C16H32O3  C16H35NO2  C18H28O2  C18H30O2  C18H30O2  C18H30O2  C18H30O2  C18H32O2  C18H35NO  C18H34O2  C18H34O2  C16H30O4  C16H32O4  C18H30O3  C18H30O3  C18H30O3  C18H30O3  C18H32O3  C18H32O3  C18H32O3  C18H32O3  C18H32O3  C20H42O  C18H37NO2  C18H36O3  C18H36O3  C17H34O4  C20H34O2  C20H36O2  C18H30O4  C18H30O4  C18H30O4  C18H30O4  C19H34O3  C20H38O2  C18H32O4  C18H32O4  C18H32O4  C18H32O4  C18H34O4  C18H34O4  C18H39NO3  C20H30O3  C18H32O5  C18H34O5  C18H34O5  C20H32O4  C20H32O5  C21H36O4  C21H36O4  C21H38O4  C21H40O4  C21H20O9  C19H40NO7P  C19H40NO7P  C20H40NO7P  C20H40NO7P  C20H42NO7P  C20H42NO7P  C21H38NO7P  C21H42NO7P  C21H42NO7P  C21H44NO7P  C21H44NO7P  C22H44NO7P  C22H44NO7P  C22H46NO7P  C23H42NO7P  C23H42NO7P  C23H44NO7P  C23H44NO7P  C23H46NO7P  C23H46NO7P  C23H48NO7P  C23H48NO7P  C23H48NO7P  C23H48NO7P  C22H45O9P  C24H42NO7P  C24H46NO7P  C24H46NO7P  C24H48NO7P  C24H48NO7P  C24H50NO7P  C24H50NO7P  C25H42NO7P  C25H44NO7P  C25H44NO7P  C25H46NO7P  C25H46NO7P  C25H48NO7P  C25H48NO7P  C25H48NO7P  C25H50NO7P  C25H52NO7P  C25H52NO7P  C26H46NO7P  C26H48NO7P  C26H48NO7P  C26H50NO7P  C26H50NO7P  C26H52NO7P  C26H52NO7P  C26H54NO7P  C27H54NO7P  C27H56NO7P  C28H48NO7P  C28H50NO7P  C28H52NO7P  C28H54NO7P  C28H54NO7P  C30H50NO7P  C30H52NO7P  C30H52NO7P  C30H54NO7P  C33H56O14  C33H58O14  C33H58O14  C33H58O14 |
| Nucleotides and derivatives | Cytosine  Uracil  Thymine  5,6-Dihydro-5-methyluracil  2-Aminopurine  Adenine  Hypoxanthine  Allopurinol  1-Methyladenine  Guanine*  Isoguanine*  Xanthine  8-Azaguanine  6-Chloropurine  Lumazine  7-Methylguanine  6-Methylmercaptopurine  1-Methylxanthine  1,7-Dimethylxanthine  2'-Deoxycytidine  Thymidine  Cytidine  Cytarabine  Uridine  β-Pseudouridine  1-beta-D-Arabinofuranosylurac​il  Cordycepin (3'-Deoxyadenosine)*  2'-Deoxyadenosine*  2'-Deoxyinosine  2'-Deoxyguanosine  Adenosine  Inosine  9-(Arabinosyl)hypoxanthine  N6-methyladenosine  2'-O-Methyladenosine  8-Hydroxy-2-deoxyguanosine  Crotonoside  Xanthosine  5-Aminoimidazole ribonucleotide  5'-Deoxy-5'-(methylthio)adenosine  N7-Methylguanosine  N6-(2-Hydroxyethyl)adenosine  2-(Dimethylamino)guanosine  Cytidine 5'-monophosphate(Cytidylic acid)  Uridine 5'-monophosphate  Cyclic 3',5'-Adenylic acid  2'-Deoxyadenosine-5'-monophosphate  2'-Deoxyinosine-5'-monophosphate  Riboprine  Adenosine 5'-monophosphate  Inosine 5'-monophosphate  Isopentenyladenine-7-N-glucoside  Succinyladenosine  Ribosyladenosine  Uridine 5'-diphosphate  Citicoline  Nicotinic acid adenine dinucleotide | C4H5N3O  C4H4N2O2  C5H6N2O2  C5H8N2O2  C5H5N5  C5H5N5  C5H4N4O  C5H4N4O  C6H7N5  C5H5N5O  C5H5N5O  C5H4N4O2  C4H4N6O  C5H3ClN4  C6H4N4O2  C6H7N5O  C6H6N4S  C6H6N4O2  C7H8N4O2  C9H13N3O4  C10H14N2O5  C9H13N3O5  C9H13N3O5  C9H12N2O6  C9H12N2O6  C9H12N2O6  C10H13N5O3  C10H13N5O3  C10H12N4O4  C10H13N5O4  C10H13N5O4  C10H12N4O5  C10H12N4O5  C11H15N5O4  C11H15N5O4  C10H13N5O5  C10H13N5O5  C10H12N4O6  C8H14N3O7P  C11H15N5O3S  C11H15N5O5  C12H17N5O5  C12H17N5O5  C9H14N3O8P  C9H13N2O9P  C10H12N5O6P  C10H14N5O6P  C10H13N4O7P  C15H21N5O4  C10H14N5O7P  C10H13N4O8P  C16H23N5O5  C14H17N5O8  C15H21N5O8  C9H14N2O12P2  C14H26N4O11P2  C21H27N7O14P2 |
| Organic acids | Oxalic acid  L-Lactic Acid  Methanesulfonic acid  Succinic anhydride  3-Methacrylic acid  2-Aminoisobutyric acid  Tartronate semialdehyde  Hydroxypyruvic acid  2-Hydroxyisobutyric acid  3-Hydroxybutyric acid  Malonic acid  Creatinine  3-Methyl-2-Oxobutanoic acid  5-Aminovaleric acid  Methylmalonic acid  succinic acid  D-Erythronolactone  β-Hydroxyisovaleric acid  (R)-(-)-3-Hydroxybutyric acid methyl ester  Aminomalonic acid  2-Picolinic acid  2-Hydroxyethylphosphonic acid  Pipecolic acid  Citraconic acid  Methylenesuccinic acid  2-Methylsuccinic acid  2-Hydroxy-2-methyl-3-oxobutanoic acid  4-Hydroxy-2-Oxopentanoic Acid  Glutaric acid  Dimethylmalonic acid  Monomethyl succinate  2-Hydroxyisocaproic acid  2-Hydroxy-4-methylpentanoic acid  6-Hydroxyhexanoic acid  L-Malic acid  Urocanic acid  Muconic acid  1-Methylpiperidine-2-carboxylic acid  4-Hydroxycyclohexylcarboxylic acid  4-Guanidinobutyric acid  α-Ketoglutaric acid  2-Acetyl-2-Hydroxybutanoic Acid  2-Methylglutaric acid  Adipic Acid  L-Citramalic acid  2-Hydroxyglutaric Acid  3-Hydroxyglutaric acid  Benzyl acetate  (R)-(-)-2-Phenylpropionic Acid  2-Hydroxyphenylacetic acid  cis-Citral  D-(-)-Mandelic acid  2-Phosphoglycolate  Allantoin  5-Acetamidopentanoic Acid  2-Propylsuccinic acid  Pimelic acid  Sodium Valproate  Piperonylic acid  3-Hydroxymandelate  1-Naphthoic acid  9-Oxononanoic acid  Decanoic acid  Trans-Citridic acid  Shikimic acid  2-Methyl-3-oxoadipic acid  2-Oxoheptanedionic acid  Suberic Acid  2-Isopropylmalic Acid*  2-Propylmalic Acid*  1-Naphthylacetic acid  1-Hydroxy-2-Naphthoate  Azelaic acid  Isocitric Acid*  Citric Acid*  Quinic Acid  Sebacate  4,8-Dihydroxyquinoline-2-carboxylic acid  Lipoic acid  DL-Benzylsuccinic acid  Jasmonic acid  Fosfosal  2,4-Dichlorophenoxyacetic Acid  Methyl jasmonate  Methyl dihydrojasmonate  Abscisic acid  2-Hydroxyhexadecanoic acid  Triethyl citrate  Argininosuccinic acid | C2H2O4  C3H6O3  CH4O3S  C4H4O3  C5H8O2  C4H9NO2  C3H4O4  C3H4O4  C4H8O3  C4H8O3  C3H4O4  C4H7N3O  C5H8O3  C5H11NO2  C4H6O4  C4H6O4  C4H6O4  C5H10O3  C5H10O3  C3H5NO4  C6H5NO2  C2H7O4P  C6H11NO2  C5H6O4  C5H6O4  C5H8O4  C5H8O4  C5H8O4  C5H8O4  C5H8O4  C5H8O4  C6H12O3  C6H12O3  C6H12O3  C4H6O5  C6H6N2O2  C6H6O4  C7H13NO2  C7H12O3  C5H11N3O2  C5H6O5  C6H10O4  C6H10O4  C6H10O4  C5H8O5  C5H8O5  C5H8O5  C9H10O2  C9H10O2  C8H8O3  C10H16O  C8H8O3  C2H5O6P  C4H6N4O3  C7H13NO3  C7H12O4  C7H12O4  C8H15NaO2  C8H6O4  C8H8O4  C11H8O2  C9H16O3  C10H20O2  C6H6O6  C7H10O5  C7H10O5  C7H10O5  C8H14O4  C7H12O5  C7H12O5  C12H10O2  C11H8O3  C9H16O4  C6H8O7  C6H8O7  C7H12O6  C10H18O4  C10H7NO4  C8H14O2S2  C11H12O4  C12H18O3  C7H7O6P  C8H6Cl2O3  C13H20O3  C13H22O3  C15H20O4  C16H32O3  C12H20O7  C10H18N4O6 |
| Others | 2,3-Dihydroxypropanal  3-Methyl-1-pentanol  Styrene  Erythrose  D-(-)-Threose  3-Methylbenzaldehyde  Nicotinamide  D-Threitol  Isonicotinic acid*  Nicotinic acid (Vitamin B3)*  (E)-cinnamamide  2-Dehydro-3-deoxy-L-arabinonate  D-Ribose*  D-Arabinose*  L-Xylose*  Ribitol*  D-Arabitol*  Xylitol*  Orotic acid  (R)-Citronellol  1-Decanol*  2-Decanol*  1,6-anhydro-β-D-glucose  4-hydroxyphenyl acrylaldehyde  1,5-Anhydro-D-glucitol  D-Xylonic acid  Pyridoxal  Norepinephrine  Pyridoxine  1,2-Decanediol  D-Glucurono-6,3-lactone  3,7-Dihydroxychromen-4-one  D-Glucono-1,5-lactone  L-Gulono-1,4-Lactone  D-Glucosamine  D-Galactose*  D-Fructose*  Inositol*  D-Glucose*  D-Mannose*  Dulcitol*  D-Sorbitol*  Allitol*  4-Pyridoxic acid  Ginkgotoxin (4-Methoxypyridoxine)  3-Phospho-D-glyceric acid  Eucommiol  D-Galacturonic acid  Gluconic acid  D-Erythrose-4-phosphate  D-Panthenol  D-Galactaric acid  D-Saccharic acid  D-Pantothenic Acid  N-Acetyl-D-glucosamine  Biotin  Nicotinate D-ribonucleoside  D-Fructose 6-phosphate  Sorbitol-6-phosphate  Vidarabine  Glucarate O-Phosphoric acid  D-Sedoheptuiose 7-phosphate  (+)-cis-Abienol  1-(sn-Glycero-3-phospho)-1D-myo-inositol  Melibiose*  D-Maltose*  Galactinol*  D-Trehalose*  D-Sucrose*  Isomaltulose*  Lactobiose*  Lactitol  Riboflavin (Vitamin B2)  Trehalose 6-phosphate  Maltotriose*  D-Panose*  D-Melezitose*  Raffinose*  Dehydrodiconiferyl alcohol-4-O-glucoside  D(+)-Melezitose O-rhamnoside  Stachyose*  D-Maltotetraose*  Nystose*  Maltopentaose | C3H6O3  C6H14O  C8H8  C4H8O4  C4H8O4  C8H8O  C6H6N2O  C4H10O4  C6H5NO2  C6H5NO2  C9H9NO  C5H8O5  C5H10O5  C5H10O5  C5H10O5  C5H12O5  C5H12O5  C5H12O5  C5H4N2O4  C10H20O  C10H22O  C10H22O  C6H10O5  C9H8O3  C6H12O5  C5H10O6  C8H9NO3  C8H11NO3  C8H11NO3  C10H22O2  C6H8O6  C9H6O4  C6H10O6  C6H10O6  C6H13NO5  C6H12O6  C6H12O6  C6H12O6  C6H12O6  C6H12O6  C6H14O6  C6H14O6  C6H14O6  C8H9NO4  C9H13NO3  C3H7O7P  C9H16O4  C6H10O7  C6H12O7  C4H9O7P  C9H19NO4  C6H10O8  C6H10O8  C9H17NO5  C8H15NO6  C10H16N2O3S  C11H14NO6+  C6H13O9P  C6H15O9P  C10H13N5O4  C6H11PO11  C7H15O10P  C20H34O  C9H19O11P  C12H22O11  C12H22O11  C12H22O11  C12H22O11  C12H22O11  C12H22O11  C12H22O11  C12H24O11  C17H20N4O6  C12H23O14P  C18H32O16  C18H32O16  C18H32O16  C18H32O16  C26H32O11  C24H42O20  C24H42O21  C24H42O21  C24H42O21  C30H52O26 |
| Phenolic acids | Phenol  benzaldehyde  4-Methylphenol  Phenylacetaldehyde  2-Hydroxybenzaldehyde (Salicylaldehyde)  4-Hydroxybenzaldehyde  benzoic Acid  3-(Hydroxymethyl)phenol  Phenyl acetate  2-Methylbenzoic Acid  4-Hydroxyacetophenone  Anthranilic Acid  2,5-Dihydroxybenzaldehyde  Protocatechualdehyde  Salicylic acid  4-Hydroxybenzoic Acid  Tyrosol  2-Nitrophenol  2-Naphthol*  1-Naphthol*  cinnamic acid  Hydrocinnamic acid  Paroxypropione  3,4-Dimethylbenzoic Acid  Methyl Anthranilate  3-MethylSalicylic acid  3-hydroxyphenylacetic acid  Anisic acid  p-Hydroxyphenyl acetic acid  Phenoxyacetic acid  Vanillin  Isovanillin  2,5-Dihydroxyacetophenone  3-(4-Hydroxyphenyl)-1-propanol  3-AminoSalicylic acid  2,3-Dihydroxybenzoic Acid*  2,5-Dihydroxybenzoic Acid; Gentisic Acid*  3,4-Dihydroxybenzoic Acid (Protocatechuic acid)*  Hydroxytyrosol  4-NitroCatechol  (E)-3-(3,4-dihydroxyphenyl)acrylaldehyde  p-Coumaric acid  3-Hydroxycinnamic acid*  2-Hydroxycinnamic acid*  IsoEugenol  2-(Formylamino)benzoic Acid  Ethylsalicylate  Ethylparaben  3-(4-Hydroxyphenyl)-propionic acid  2,6-Dimethoxybenzaldehyde  4'-Hydroxy-3'-methoxyacetophenone (Acetovanillone)  3-Hydroxyphenylacetic Acid Methyl Ester  2-Amino-3-methoxybenzoic Acid  3,4-Dihydroxybenzeneacetic acid  Homogentisic acid  Vanillic acid  Methyl 3,4-dihydroxybenzoate  Phloracetophenone  Isovanillic acid  2-Methoxy-5-nitrophenol  Coniferaldehyde  p-Coumaric acid methyl ester  4-Methoxycinnamic acid  Caffeic acid  coniferyl alcohol  Propylparaben  5-AcetylSalicylic acid  Hydroxyphenyllactic acid  Methyl 2,4-dihydroxyphenylacetate  Syringaldehyde; 4-Hydroxy-3,5-Dimethoxybenzaldehyde  Homovanillic acid; 4-Hydroxy-3-methoxyphenylacetic acid  2,6-Dimethoxybenzoic Acid  Methyl vanillate  2,4-Dinitrophenol  3-O-Methylgallic acid  p-Coumaric acid ethyl ester  Ferulic acid*  Methyl caffeate  Isoferulic Acid*  Dihydroferulic Acid  3,4-Dimethoxyphenyl acetic acid  Orsellinic acid ethyl ester  4-Hydroxy-3-methoxymandelate  syringic acid  2,6-Di-tert-butylphenol  3-[(1-Carboxyvinyl)oxy]benzoic Acid  Sinapinaldehyde  Methyl ferulate  Ethyl caffeate  Ferulic acid methyl ester  Elemicin  Vanillyl Butyl Ether  Methyl syringate  alpha-hexylcinnamaldehyde  Ethyl ferulate  Stilbostemin B  Benzoyltartaric acid  1-Feruloyl-sn-glycerol  Arbutin  Phloroglucinol-1-O-β-D-glucopyranoside  1-O-Salicyl-D-glucose  Glucosyloxybenzoic Acid  3-methoxy-5-hydroxy-1-O-β-D-glucopyranoside  3-Methoxy-4-hydroxyphenol-1-O-β-D-glucoside (Tachioside)  1-O-Gentisoyl-D-glucoside  Protocatechuic acid-4-O-glucoside  5-(2-Hydroxyethyl)-2-O-glucosylphenol  Erianin  3-Hydroxy-4-isopropylbenzylalcohol-3-O-glucoside  3,4,5-Trimethoxyphenyl-1-O-Glucoside  Gallic acid-4-O-glucoside  2-(3,4-dihydroxyphenyl)ethanediol 1-O-β-D-glucopyranoside  Koaburaside  3-O-p-Coumaroylquinic acid*  5-O-p-Coumaroylquinic acid*  Sinapoyl malate  P-Methoyxcinnamate glucoside  6-O-Caffeoyl-D-glucose  coniferyl alcohol-4-O-glucoside (Coniferin)  Dihydrocaffeoylglucose  Syringaldehyde-4-O-glucoside  Neochlorogenic acid (5-O-caffeoylquinic acid)*  Cryptochlorogenic acid (4-O-caffeoylquinic acid)*  Chlorogenic acid (3-O-caffeoylquinic acid)*  1-O-Feruloyl-D-Glucose  Rosmarinic acid  Glucosyringic acid  3-O-Feruloylquinic acid  Chlorogenic acid methyl ester  Trihydroxycinnamoylquinic acid  Feruloyl syringic acid  1-O-Sinapoyl-D-glucose  5'-Glucosyloxyjasmanic acid  Salireposide  2-Hydroxyphenol-1-O-glucosyl(6→1)rhamnoside  6-O-Glucosyl-caffeoylbenzoic Acid  cinnamoylferuloyltartaric acid  6-O-Glucosyl-feruloylbenzoic Acid  p-Coumaroylferuloyltartaric acid  Dicaffeoylshikimic acid  Isochlorogenic acid A*  3,5-Dicaffeoylquinic acid*  Isochlorogenic acid B*  4,5-Dicaffeoylquinic acid*  1,3-Dicaffeoylquinic acid*  Rosmarinic acid-3'-O-glucoside  3,5-O-Dicaffeoylquinic acid Methyl Ester  3,4,5-Tricaffeoylquinic acid | C6H6O  C7H6O  C7H8O  C8H8O  C7H6O2  C7H6O2  C7H6O2  C7H8O2  C8H8O2  C8H8O2  C8H8O2  C7H7NO2  C7H6O3  C7H6O3  C7H6O3  C7H6O3  C8H10O2  C6H5NO3  C10H8O  C10H8O  C9H8O2  C9H10O2  C9H10O2  C9H10O2  C8H9NO2  C8H8O3  C8H8O3  C8H8O3  C8H8O3  C8H8O3  C8H8O3  C8H8O3  C8H8O3  C9H12O2  C7H7NO3  C7H6O4  C7H6O4  C7H6O4  C8H10O3  C6H5NO4  C9H8O3  C9H8O3  C9H8O3  C9H8O3  C10H12O2  C8H7NO3  C9H10O3  C9H10O3  C9H10O3  C9H10O3  C9H10O3  C9H10O3  C8H9NO3  C8H8O4  C8H8O4  C8H8O4  C8H8O4  C8H8O4  C8H8O4  C7H7NO4  C10H10O3  C10H10O3  C10H10O3  C9H8O4  C10H12O3  C10H12O3  C9H8O4  C9H10O4  C9H10O4  C9H10O4  C9H10O4  C9H10O4  C9H10O4  C6H4N2O5  C8H8O5  C11H12O3  C10H10O4  C10H10O4  C10H10O4  C10H12O4  C10H12O4  C10H12O4  C9H10O5  C9H10O5  C14H22O  C10H8O5  C11H12O4  C11H12O4  C11H12O4  C11H12O4  C12H16O3  C12H18O3  C10H12O5  C15H20O  C12H14O4  C15H16O2  C11H10O7  C13H16O6  C12H16O7  C12H16O8  C13H16O8  C13H16O8  C13H18O8  C13H18O8  C13H16O9  C13H16O9  C14H20O8  C18H22O5  C16H24O7  C15H22O8  C13H16O10  C14H20O9  C14H20O9  C16H18O8  C16H18O8  C15H16O9  C16H20O8  C15H18O9  C16H22O8  C15H20O9  C15H20O9  C16H18O9  C16H18O9  C16H18O9  C16H20O9  C18H16O8  C15H20O10  C17H20O9  C17H20O9  C16H20O10  C19H18O8  C17H22O10  C18H28O9  C20H22O9  C18H26O11  C22H22O10  C23H20O10  C23H24O10  C23H20O11  C25H22O11  C25H24O12  C25H24O12  C25H24O12  C25H24O12  C25H24O12  C24H26O13  C26H26O12  C34H30O15 |
| Terpenoids | Blumenol C  Ambolic acid  2-Hydroxyoleanolic acid  3,24-Dihydroxy-17,21-semiacetal-12(13)oleanolic fruit  Echinocystic acid  2,3-dihydroxy-12-ursen-28-oic acid  Maslinic acid  Alphitolic acid  Corosolic acid  Swinhoeic acid  1-Oxo-Siaresinolic acid  Quillaic acid  2α,3α,19α,23-tetrahydroxy-12-ursen-28-oic acid  2α,3β,19α,23-Tetrahydroxyolean-12-en-28-oic acid  2α,3β,19α,23-Tetrahydroxyurs-12-en-28-oic acid  1β,2α,3α,19α,23-Pentahydroxyurs-12-en-28-oic acid | C13H22O2  C31H50O3  C30H48O4  C30H48O4  C30H48O4  C30H48O4  C30H48O4  C30H48O4  C30H48O4  C30H46O5  C30H46O5  C30H46O5  C30H48O6  C30H48O6  C30H48O6  C30H48O7 |

**Table S2:** Eighty-six key differential metabolites in seedling stage.

| Class | Compounds | KEGG ID |
| --- | --- | --- |
| Alkaloids | Methyl nicotinate  Spermine  2-(Acetylamino)-3-phenyl-2-propenoic acid  N-Oleoylethanolamine | C01004  C00750  --  C20792 |
| Amino acids and derivatives | Cycloleucine  L-Methionine Sulfoxide  N-Acetyl-L-leucine  Cyclo(Ser-Pro)  N-Acetyl-L-Glutamine  N6-Acetyl-L-lysine  Trimethyllysine  L-Isoleucyl-L-Aspartate | C03969  C02989  C02710  --  --  C02727  C03793  -- |
| Flavonoids | Norwogonin | C10113 |
| Lignans and Coumarins | Xanthotoxol | C00841 |
| Lipids | Palmitoleic Acid  9-Oxo-12Z-Octadecenoic acid  15(R)-Hydroxylinoleic Acid  4-Hydroxysphinganine  9,10-Dihydroxy-12,13-epoxyoctadecanoic acid  LysoPE 14:0*  LysoPE 14:0(2n isomer)*  LysoPE 15:1(2n isomer)*  LysoPE 16:3  LysoPE 17:1(2n isomer)*  LysoPE 17:1*  LysoPC 14:0  LysoPE 18:2(2n isomer)*  LysoPE 18:0(2n isomer)*  LysoPC 16:2*  LysoPC 16:1(2n isomer)*  LysoPC 16:1*  LysoPC 16:0(2n isomer)*  LysoPC 16:0*  LysoPC 19:1  LysoPC 19:0 | C08362  --  --  C12144  C14837  --  --  --  --  --  --  --  --  --  --  --  --  --  --  --  -- |
| Nucleotides and derivatives | 5,6-Dihydro-5-methyluracil  1-beta-D-Arabinofuranosylurac​il | C00906  C16908 |
| Organic acids | Methanesulfonic acid  Malonic acid  D-Erythronolactone  2-Picolinic acid  Pipecolic acid  2-Hydroxy-4-methylpentanoic acid  Urocanic acid  L-Citramalic acid  2-Hydroxyglutaric Acid  Allantoin  2-Oxoheptanedionic acid  2-Isopropylmalic Acid*  2-Propylmalic Acid*  1-Hydroxy-2-Naphthoate  4,8-Dihydroxyquinoline-2-carboxylic acid  DL-Benzylsuccinic acid  Fosfosal  Abscisic acid  Argininosuccinic acid | C11145  C00383  --  C10164  C00408  --  C00785  C02614  C03196  C01551  C16588  C02504  C05994  C03203  C02470  --  --  C06082  C03406 |
| Others | Erythrose  D-(-)-Threose  D-Threitol  Isonicotinic acid*  Nicotinic acid (Vitamin B3)*  2-Dehydro-3-deoxy-L-arabinonate  D-Glucono-1,5-lactone  L-Gulono-1,4-Lactone  D-Saccharic acid  D-Sucrose*  D-Panose* | C01796  C06463  C16884  C07446  C00253  C00684  C00198  C01040  C00818  C00089  C00713 |
| Phenolic acids | 4-Methylphenol  Phenylacetaldehyde  4-Hydroxyacetophenone  2-Naphthol*  1-Naphthol*  cinnamic acid  Anisic acid  3-(4-Hydroxyphenyl)-1-propanol  Hydroxytyrosol  p-Coumaric acid  IsoEugenol  2,6-Dimethoxybenzaldehyde  3-Hydroxyphenylacetic Acid Methyl Ester  5-AcetylSalicylic acid  p-Coumaric acid ethyl ester  syringic acid  Ethyl ferulate  Koaburaside  Salireposide | C01468  C00601  C10700  C11713  C11714  C00423  C02519  --  --  C00811  C10469  --  --  --  --  C10833  --  --  -- |

**Table S3:** One hundred and thirty-eight key differential metabolites in maturity.

| Class | Compounds | KEGG ID |
| --- | --- | --- |
| Alkaloids | Phenethylamine  N-Acetylcadaverine  N'-p-Coumaroylagmatine-glucoside  Diferuloylcadaverine | C05332  --  --  -- |
| Amino acids and derivatives | L-Proline  Cycloleucine  N6-Acetyl-L-lysine  L-Glycyl-L-isoleucine*  N-Glycyl-L-leucine*  L-Homocitrulline  Cyclo(Pro-Leu)  N'-Formylkynurenine  N-Acetyl-L-Tryptophan | C00148  C03969  C02727  --  C02155  C02427  --  C02700  -- |
| Flavonoids | Quercetin-3-O-Sulfonate  Quercetin-3-O-glucoside (Isoquercitrin)  Luteolin-7-O-neohesperidoside (lonicerin)  Quercetin-3-O-(4''-O-glucosyl)rhamnoside | C00616  C05623  C12630  C17563 |
| Lignans and Coumarins | Bergaptol  scopoletin-7-O-glucoside (Scopolin)  Matairesinol  Arctigenin  lariciresinol-4'-O-glucoside | C00758  C01527  C10682  C10545  -- |
| Lipids | 10-Heptadecenoic Acid  α-Linolenic Acid*  γ-Linolenic Acid*  Linoleic acid  Oleic acid  2R-hydroxy-9Z,12Z,15Z-octadecatrienoic acid  17-Hydroxylinolenic acid  13S-Hydroxy-9Z,11E,15Z-octadecatrienoic acid  9S-Hydroxy-10E,12Z-octadecadienoic acid  13(S)-HODE;13(S)-Hydroxyoctadeca-9Z,11E-dienoic acid  4-Hydroxysphinganine  12-Oxo-5,8,10,14-eicosatetraenoic acid  2-α-Linolenoyl-glycerol*  1-α-Linolenoyl-glycerol*  2-Linoleoylglycerol*  1-Oleoyl-Sn-Glycerol  LysoPE 14:0*  LysoPE 14:0(2n isomer)*  LysoPE 15:1*  LysoPE 15:1(2n isomer)*  LysoPE 15:0(2n isomer)*  LysoPE 16:1*  LysoPE 16:1(2n isomer)*  LysoPE 16:0(2n isomer)*  LysoPE 16:0*  LysoPE 17:1(2n isomer)*  LysoPE 17:1*  LysoPC 14:0  LysoPE 18:3(2n isomer)*  LysoPE 18:3*  LysoPE 18:2(2n isomer)*  LysoPE 18:2*  LysoPE 18:1(2n isomer)*  LysoPE 18:1*  LysoPE 18:0(2n isomer)*  LysoPC 15:0(2n isomer)*  LysoPC 16:2(2n isomer)*  LysoPC 16:1(2n isomer)*  LysoPC 16:0(2n isomer)*  LysoPC 16:0*  LysoPE 20:5  LysoPE 20:4(2n isomer)*  LysoPE 20:4*  LysoPE 20:3(2n isomer)*  LysoPE 20:3*  LysoPC 17:2  LysoPE 20:2*  LysoPE 20:2(2n isomer)*  LysoPC 17:1  LysoPC 17:0(2n isomer)*  LysoPC 18:4  LysoPC 18:3(2n isomer)*  LysoPC 18:3*  LysoPC 18:2*  LysoPC 18:2(2n isomer)*  LysoPC 18:1(2n isomer)*  LysoPC 18:0(2n isomer)*  LysoPC 19:1  LysoPC 20:5  LysoPC 20:4  LysoPC 20:3  LysoPC 20:2(2n isomer)*  LysoPC 20:2*  LysoPC 22:6  LysoPC 22:5*  LysoPC 22:5(2n isomer)*  LysoPC 22:4  1-Linolenoyl-rac-glycerol-diglucoside  1-Linoleoylglycerol-2,3-di-O-glucoside*  1-Linoleoyl-sn-glycerol-diglucoside  2-Linoleoylglycerol-1,3-di-O-glucoside* | --  C06427  C06426  C01595  C00712  C16342  C16346  C16316  C14767  C14762  C12144  C14807  --  --  --  --  --  --  --  --  --  --  --  --  --  --  --  --  --  --  --  --  --  --  --  --  --  --  --  --  --  --  --  --  --  --  --  --  --  --  --  --  --  --  --  --  --  --  --  --  --  --  --  --  --  --  --  --  --  --  -- |
| Nucleotides and derivatives | Inosine  9-(Arabinosyl)hypoxanthine  N6-methyladenosine  Xanthosine  2'-Deoxyinosine-5'-monophosphate  Succinyladenosine | C00294  --  --  C01762  C06196  -- |
| Organic acids | 2-Picolinic acid  Pipecolic acid  D-(-)-Mandelic acid  1-Hydroxy-2-Naphthoate  Isocitric Acid*  Jasmonic acid | C10164  C00408  --  C03203  C00311  C08491 |
| Others | D-Threitol  Isonicotinic acid*  Nicotinic acid (Vitamin B3)*  D-Arabinose*  D-Galactose*  D-Fructose*  D-Glucose*  D-Mannose*  D-Erythrose-4-phosphate  Lactitol  Dehydrodiconiferyl alcohol-4-O-glucoside  D(+)-Melezitose O-rhamnoside | C16884  C07446  C00253  C00216  C00124  C05003  C00031  C00159  C00279  --  --  -- |
| Phenolic acids | Phenol  Phenyl acetate  2-Methylbenzoic Acid  Methyl Anthranilate  Phenoxyacetic acid  Syringaldehyde; 4-Hydroxy-3,5-Dimethoxybenzaldehyde  2,4-Dinitrophenol  Sinapinaldehyde  Methyl ferulate  Ferulic acid methyl ester  Vanillyl Butyl Ether  Benzoyltartaric acid  3-Methoxy-4-hydroxyphenol-1-O-β-D-glucoside (Tachioside)  3-Hydroxy-4-isopropylbenzylalcohol-3-O-glucoside  P-Methoyxcinnamate glucoside  Syringaldehyde-4-O-glucoside  6-O-Glucosyl-caffeoylbenzoic Acid  6-O-Glucosyl-feruloylbenzoic Acid  Rosmarinic acid-3'-O-glucoside | C00146  C00548  C07215  C20634  C02181  --  C02496  C05610  --  --  --  --  --  --  --  --  --  --  -- |
| Terpenoids | 1-Oxo-Siaresinolic acid  1β,2α,3α,19α,23-Pentahydroxyurs-12-en-28-oic acid | --  -- |
