# Supplementary material for: Nitrogen deposition further increases Ambrosia trifida root exudate invasiveness under global warming
Source: Environ Monit Assess. 2023 May 30;195(6):759. doi: 10.1007/s10661-023-11380-w (PMC10229694; doi:10.1007/s10661-023-11380-w)
Supplement: Supplementary file 2 — Supplementary file2 (DOCX 99 KB) [file 10661_2023_11380_MOESM2_ESM.docx]

| Group | Compounds | Class | Fold_Change | VIP | Type |
| --- | --- | --- | --- | --- | --- |
| SC vs SW | L-Proline | Amino acids and derivatives | 2.353 | 1.132 | up |
|  | L-Methionine Sulfoxide | Amino acids and derivatives | 666.736 | 1.641 | up |
|  | N-Acetyl-L-leucine | Amino acids and derivatives | 3.302 | 1.135 | up |
|  | Cyclo(Ser-Pro) | Amino acids and derivatives | 1142.641 | 2.298 | up |
|  | N-Acetyl-L-Glutamine | Amino acids and derivatives | 5.579 | 1.734 | up |
|  | N6-Acetyl-L-lysine | Amino acids and derivatives | 5.669 | 1.115 | up |
|  | N-Glycyl-L-leucine* | Amino acids and derivatives | 3.447 | 1.002 | up |
|  | Trimethyllysine | Amino acids and derivatives | 7.399 | 1.281 | up |
|  | L-Homocitrulline | Amino acids and derivatives | 8.417 | 1.761 | up |
|  | N-Acetyl-L-tyrosine | Amino acids and derivatives | 3.852 | 1.670 | up |
|  | L-Prolyl-L-Leucine | Amino acids and derivatives | 9.959 | 1.204 | up |
|  | L-Alanyl-L-Phenylalanine | Amino acids and derivatives | 2.664 | 1.013 | up |
|  | L-Isoleucyl-L-Aspartate | Amino acids and derivatives | 5.699 | 1.661 | up |
|  | L-Phenylalanyl-L-phenylalanine | Amino acids and derivatives | 2.480 | 1.018 | up |
|  | 4-Methylphenol | Phenolic acids | 1562.013 | 1.605 | up |
|  | Phenylacetaldehyde | Phenolic acids | 2.016 | 1.199 | up |
|  | 4-Hydroxyacetophenone | Phenolic acids | 2.177 | 1.376 | up |
|  | cinnamic acid | Phenolic acids | 3.230 | 1.023 | up |
|  | 3-(4-Hydroxyphenyl)-1-propanol | Phenolic acids | 1867.425 | 1.604 | up |
|  | 3-AminoSalicylic acid | Phenolic acids | 2.603 | 1.907 | up |
|  | Hydroxytyrosol | Phenolic acids | 2.256 | 1.199 | up |
|  | p-Coumaric acid | Phenolic acids | 1781.613 | 2.305 | up |
|  | 3-(4-Hydroxyphenyl)-propionic acid | Phenolic acids | 4.058 | 1.068 | up |
|  | 2,6-Dimethoxybenzaldehyde | Phenolic acids | 4.313 | 1.075 | up |
|  | 5-AcetylSalicylic acid | Phenolic acids | 2.931 | 1.258 | up |
|  | p-Coumaric acid ethyl ester | Phenolic acids | 2.361 | 1.151 | up |
|  | syringic acid | Phenolic acids | 2.116 | 1.194 | up |
|  | Vanillyl Butyl Ether | Phenolic acids | 530.482 | 2.302 | up |
|  | Ethyl ferulate | Phenolic acids | 2.308 | 1.182 | up |
|  | Koaburaside | Phenolic acids | 2.392 | 1.090 | up |
|  | 5-O-p-Coumaroylquinic acid* | Phenolic acids | 613.567 | 1.605 | up |
|  | Neochlorogenic acid (5-O-caffeoylquinic acid)* | Phenolic acids | 2.071 | 1.010 | up |
|  | Salireposide | Phenolic acids | 15.542 | 1.872 | up |
|  | 6-O-Glucosyl-feruloylbenzoic Acid | Phenolic acids | 165.881 | 1.038 | up |
|  | Isochlorogenic acid B* | Phenolic acids | 257.763 | 1.038 | up |
|  | 5,6-Dihydro-5-methyluracil | Nucleotides and derivatives | 542.741 | 1.038 | up |
|  | Xanthine | Nucleotides and derivatives | 3.388 | 1.095 | up |
|  | 1-beta-D-Arabinofuranosylurac il | Nucleotides and derivatives | 3.163 | 1.619 | up |
|  | Xanthosine | Nucleotides and derivatives | 4.153 | 1.057 | up |
|  | 5-Aminoimidazole ribonucleotide | Nucleotides and derivatives | 3.581 | 1.276 | up |
|  | Uridine 5'-monophosphate | Nucleotides and derivatives | 2.989 | 1.221 | up |
|  | Apigenin-6,8-di-C-glucoside (Vicenin-2) | Flavonoids | 193.838 | 1.605 | up |
|  | Xanthotoxol | Lignans and Coumarins | 15.411 | 1.527 | up |
|  | O-Feruloyl 3-hydroxycoumarin | Lignans and Coumarins | 1008.299 | 1.592 | up |
|  | Isofraxidin-7-O-glucoside | Lignans and Coumarins | 87.473 | 1.038 | up |
|  | Isonicotinic acid* | Others | 3.232 | 1.315 | up |
|  | Nicotinic acid (Vitamin B3)* | Others | 3.123 | 1.256 | up |
|  | 2-Dehydro-3-deoxy-L-arabinonate | Others | 2.908 | 1.560 | up |
|  | Orotic acid | Others | 21.658 | 1.826 | up |
|  | D-Xylonic acid | Others | 2.648 | 1.009 | up |
|  | L-Gulono-1,4-Lactone | Others | 2.298 | 1.615 | up |
|  | D-Erythrose-4-phosphate | Others | 3.064 | 1.557 | up |
|  | D-Sucrose* | Others | 2.469 | 1.173 | up |
|  | Choline | Alkaloids | 2.765 | 1.499 | up |
|  | Methyl nicotinate | Alkaloids | 2.582 | 1.516 | up |
|  | Histidinol | Alkaloids | 2.230 | 1.602 | up |
|  | Quinolinic acid | Alkaloids | 2.378 | 1.122 | up |
|  | 2-(Acetylamino)-3-phenyl-2-propenoic acid | Alkaloids | 4.052 | 1.407 | up |
|  | N-Oleoylethanolamine | Alkaloids | 5.620 | 1.451 | up |
|  | p-Coumaroylferuloylcadaverine | Alkaloids | 2.155 | 1.221 | up |
|  | Blumenol C | Terpenoids | 892.430 | 1.038 | up |
|  | 2α,3α,19α,23-tetrahydroxy-12-ursen-28-oic acid | Terpenoids | 529.981 | 1.082 | up |
|  | 3-Methacrylic acid | Organic acids | 935.711 | 1.675 | up |
|  | Hydroxypyruvic acid | Organic acids | 79.275 | 1.013 | up |
|  | Malonic acid | Organic acids | 4.354 | 1.130 | up |
|  | succinic acid | Organic acids | 3455.873 | 1.605 | up |
|  | D-Erythronolactone | Organic acids | 2.125 | 1.484 | up |
|  | β-Hydroxyisovaleric acid | Organic acids | 3.164 | 1.144 | up |
|  | (R)-(-)-3-Hydroxybutyric acid methyl ester | Organic acids | 33.891 | 1.123 | up |
|  | 2-Picolinic acid | Organic acids | 3.673 | 1.624 | up |
|  | Pipecolic acid | Organic acids | 2.218 | 1.036 | up |
|  | Methylenesuccinic acid | Organic acids | 450.675 | 2.281 | up |
|  | 2-Hydroxyisocaproic acid | Organic acids | 2.081 | 1.253 | up |
|  | 2-Hydroxy-4-methylpentanoic acid | Organic acids | 2.172 | 1.345 | up |
|  | 6-Hydroxyhexanoic acid | Organic acids | 2.709 | 1.176 | up |
|  | Urocanic acid | Organic acids | 5.811 | 1.687 | up |
|  | α-Ketoglutaric acid | Organic acids | 677.991 | 1.082 | up |

**TableS2**: Differential metabolites of different treatment groups

| 2-Methylglutaric acid | Organic acids | 2.042 | 1.109 | up |
| --- | --- | --- | --- | --- |
| L-Citramalic acid | Organic acids | 3.309 | 1.146 | up |
| 2-Hydroxyphenylacetic acid | Organic acids | 46.476 | 1.539 | up |
| cis-Citral | Organic acids | 39.912 | 1.082 | up |
| Allantoin | Organic acids | 3.362 | 2.011 | up |
| 5-Acetamidopentanoic Acid | Organic acids | 788.356 | 1.603 | up |
| 2-Propylsuccinic acid | Organic acids | 33419.815 | 2.210 | up |
| Pimelic acid | Organic acids | 43.013 | 1.430 | up |
| 9-Oxononanoic acid | Organic acids | 2.235 | 1.012 | up |
| 2-Methyl-3-oxoadipic acid | Organic acids | 1381.869 | 1.675 | up |
| 2-Oxoheptanedionic acid | Organic acids | 1495.998 | 2.311 | up |
| 2-Isopropylmalic Acid* | Organic acids | 5.253 | 1.676 | up |
| 2-Propylmalic Acid* | Organic acids | 7.261 | 1.724 | up |
| Sebacate | Organic acids | 790.944 | 1.606 | up |
| 4,8-Dihydroxyquinoline-2-carboxylic acid | Organic acids | 367.185 | 1.038 | up |
| DL-Benzylsuccinic acid | Organic acids | 4.316 | 1.675 | up |
| Fosfosal | Organic acids | 4413.351 | 1.589 | up |
| Abscisic acid | Organic acids | 2.264 | 1.078 | up |
| Argininosuccinic acid | Organic acids | 328.372 | 1.676 | up |
| 2-Dodecenedioic acid | Lipids | 1015.871 | 2.285 | up |
| Palmitoleic Acid | Lipids | 8.008 | 1.786 | up |
| Punicic acid (9Z,11E,13Z-octadecatrienoic acid) | Lipids | 159.081 | 1.038 | up |
| Linoleic acid | Lipids | 2.005 | 1.067 | up |
| 13-KODE; (9Z,11E)-13-Oxooctadeca-9,11-dienoic acid | Lipids | 6.340 | 1.696 | up |
| 9-Oxo-12Z-Octadecenoic acid | Lipids | 2.492 | 1.352 | up |
| 15(R)-Hydroxylinoleic Acid | Lipids | 2.403 | 1.320 | up |
| 1-Eicosanol | Lipids | 6.452 | 1.437 | up |
| 3-Dehydrosphinganine | Lipids | 23.890 | 1.607 | up |
| Eicosadienoic acid | Lipids | 2.160 | 1.168 | up |
| 9-Hydroperoxy-9Z,11E-Octadecadienoic Acid | Lipids | 3.492 | 1.216 | up |
| Hydroxy ricinoleic acid | Lipids | 683.230 | 1.038 | up |
| 4-Hydroxysphinganine | Lipids | 5.219 | 1.774 | up |
| 9,12,13-Trihydroxy-10,15-octadecadienoic acid | Lipids | 2.663 | 1.164 | up |
| 9,10,13-Trihydroxy-11-Octadecenoic Acid | Lipids | 2.320 | 1.117 | up |
| 9,10-Dihydroxy-12,13-epoxyoctadecanoic acid | Lipids | 2694.621 | 1.601 | up |
| LysoPE 14:0* | Lipids | 3.511 | 1.081 | up |
| LysoPE 15:1(2n isomer)* | Lipids | 2.455 | 1.220 | up |
| LysoPE 17:1(2n isomer)* | Lipids | 3.103 | 1.169 | up |
| LysoPE 17:1* | Lipids | 2.633 | 1.059 | up |
| LysoPC 14:0 | Lipids | 4.768 | 1.402 | up |
| LysoPC 16:2(2n isomer)* | Lipids | 6.827 | 1.777 | up |
| LysoPC 16:2* | Lipids | 27.991 | 1.677 | up |
| LysoPC 16:1(2n isomer)* | Lipids | 4.074 | 1.366 | up |
| LysoPC 16:1* | Lipids | 4.183 | 1.383 | up |
| LysoPC 16:0(2n isomer)* | Lipids | 4.249 | 1.762 | up |
| LysoPC 16:0* | Lipids | 3.878 | 1.692 | up |
| LysoPC 17:1 | Lipids | 2.361 | 1.138 | up |
| LysoPC 17:0* | Lipids | 93.339 | 1.038 | up |
| LysoPC 18:3* | Lipids | 2.087 | 1.631 | up |
| LysoPC 18:2* | Lipids | 2.703 | 2.091 | up |
| LysoPC 18:2(2n isomer)* | Lipids | 2.497 | 1.958 | up |
| LysoPC 18:1(2n isomer)* | Lipids | 3.355 | 1.557 | up |
| LysoPC 18:1* | Lipids | 3.358 | 1.536 | up |
| LysoPC 19:1 | Lipids | 3.599 | 2.000 | up |
| LysoPC 19:0 | Lipids | 2.881 | 1.664 | up |
| LysoPC 20:2* | Lipids | 31.247 | 1.038 | up |
| 1-Linolenoyl-rac-glycerol-diglucoside | Lipids | 428.296 | 1.082 | up |
| L-Alanine | Amino acids and derivatives | 0.013 | 1.046 | down |
| 2-Naphthol* | Phenolic acids | 0.001 | 2.302 | down |
| 1-Naphthol* | Phenolic acids | 0.001 | 2.303 | down |
| Phenoxyacetic acid | Phenolic acids | 0.346 | 1.515 | down |
| IsoEugenol | Phenolic acids | 0.035 | 1.705 | down |
| Ethylsalicylate | Phenolic acids | 0.002 | 1.072 | down |
| 3-Hydroxyphenylacetic Acid Methyl Ester | Phenolic acids | 0.320 | 1.196 | down |
| 4,5-Dicaffeoylquinic acid* | Phenolic acids | 0.004 | 1.611 | down |
| 2'-O-Methyladenosine | Nucleotides and derivatives | 0.147 | 1.397 | down |
| 2'-Deoxyinosine-5'-monophosphate | Nucleotides and derivatives | 0.107 | 2.038 | down |
| scopoletin (7-Hydroxy-5-methoxycoumarin) | Lignans and Coumarins | 0.001 | 1.072 | down |
| Matairesinol | Lignans and Coumarins | 0.182 | 1.184 | down |
| D-Threitol | Others | 0.432 | 1.739 | down |
| D-Panose* | Others | 0.002 | 2.308 | down |
| D-Maltotetraose* | Others | 0.006 | 1.072 | down |
| 2,3-dihydroxy-12-ursen-28-oic acid | Terpenoids | 0.259 | 1.218 | down |
| Methanesulfonic acid | Organic acids | 0.373 | 1.770 | down |
| (R)-(-)-2-Phenylpropionic Acid | Organic acids | 0.364 | 1.243 | down |
| 1-Hydroxy-2-Naphthoate | Organic acids | 0.392 | 2.105 | down |
| 9,12-Octadecadien-6-Ynoic Acid | Lipids | 0.011 | 1.072 | down |
| 2R-hydroxy-9Z,12Z,15Z-octadecatrienoic acid | Lipids | 0.003 | 1.072 | down |

|  | 12-Oxo-5,8,10,14-eicosatetraenoic acid | Lipids | 0.023 | 1.072 | down |
| --- | --- | --- | --- | --- | --- |
|  | LysoPE 20:4(2n isomer)* | Lipids | 0.374 | 1.028 | down |
| SW vs SWN | D-Proline betaine | Amino acids and derivatives | 5.051 | 1.156 | up |
|  | Cyclo(L-Ala-L-Pro) | Amino acids and derivatives | 2.979 | 1.976 | up |
|  | L-Tyrosine | Amino acids and derivatives | 2.414 | 1.066 | up |
|  | Cyclo(Pro-Val) | Amino acids and derivatives | 6.438 | 1.381 | up |
|  | Cyclo(Pro-Leu) | Amino acids and derivatives | 4.247 | 1.111 | up |
|  | Cyclo(D-Phe-L-Pro) | Amino acids and derivatives | 5.016 | 1.103 | up |
|  | Cyclo(Pro-Phe) | Amino acids and derivatives | 5.480 | 1.128 | up |
|  | Paroxypropione | Phenolic acids | 2.137 | 1.967 | up |
|  | Anisic acid | Phenolic acids | 2.272 | 1.328 | up |
|  | 4-Methoxycinnamic acid | Phenolic acids | 2.054 | 1.030 | up |
|  | 5-AcetylSalicylic acid | Phenolic acids | 5.494 | 1.932 | up |
|  | p-Coumaric acid ethyl ester | Phenolic acids | 2.277 | 1.512 | up |
|  | syringic acid | Phenolic acids | 5.070 | 1.278 | up |
|  | Elemicin | Phenolic acids | 2.503 | 1.197 | up |
|  | Vanillyl Butyl Ether | Phenolic acids | 2.577 | 1.194 | up |
|  | Methyl syringate | Phenolic acids | 2.144 | 2.072 | up |
|  | Erianin | Phenolic acids | 2.101 | 1.638 | up |
|  | Lumazine | Nucleotides and derivatives | 22946.296 | 1.571 | up |
|  | Inosine | Nucleotides and derivatives | 3.023 | 1.616 | up |
|  | 5'-Deoxy-5'-(methylthio)adenosine | Nucleotides and derivatives | 2.723 | 1.133 | up |
|  | 2'-Deoxyinosine-5'-monophosphate | Nucleotides and derivatives | 11.890 | 1.848 | up |
|  | Inosine 5'-monophosphate | Nucleotides and derivatives | 2.863 | 1.136 | up |
|  | Succinyladenosine | Nucleotides and derivatives | 8.646 | 1.145 | up |
|  | Nicotinic acid adenine dinucleotide | Nucleotides and derivatives | 14.279 | 1.319 | up |
|  | Pinostrobin | Flavonoids | 20.784 | 1.417 | up |
|  | 6,7-Dihydroxy-4-methylcoumarin | Lignans and Coumarins | 5.311 | 1.645 | up |
|  | Fraxetin (7,8-Dihydroxy-6-methoxycoumarin) | Lignans and Coumarins | 2.110 | 1.241 | up |
|  | Fraxidin (8-Hydroxy-6,7-dimethoxycoumarin)* | Lignans and Coumarins | 2.054 | 1.572 | up |
|  | isofraxidin* | Lignans and Coumarins | 4.428 | 1.383 | up |
|  | Matairesinol | Lignans and Coumarins | 6.473 | 1.842 | up |
|  | D-(-)-Threose | Others | 2.926 | 1.058 | up |
|  | Isonicotinic acid* | Others | 2.564 | 1.027 | up |
|  | D-Ribose* | Others | 2.511 | 1.711 | up |
|  | Xylitol* | Others | 3.709 | 1.175 | up |
|  | Inositol* | Others | 2.339 | 1.572 | up |
|  | 3-Phospho-D-glyceric acid | Others | 2.386 | 1.208 | up |
|  | D-Galacturonic acid | Others | 2.015 | 1.648 | up |
|  | D-Saccharic acid | Others | 2.551 | 1.037 | up |
|  | Biotin | Others | 4.114 | 1.800 | up |
|  | Galactinol* | Others | 3.284 | 1.548 | up |
|  | 4-Aminophenol | Alkaloids | 3.702 | 1.176 | up |
|  | 6-Hydroxynicotinic acid | Alkaloids | 3.630 | 1.148 | up |
|  | N-Oleoylethanolamine | Alkaloids | 4.127 | 1.161 | up |
|  | Ambolic acid | Terpenoids | 2.363 | 1.102 | up |
|  | 2-Aminoisobutyric acid | Organic acids | 6.785 | 1.304 | up |
|  | Urocanic acid | Organic acids | 2.343 | 1.474 | up |
|  | 4-Guanidinobutyric acid | Organic acids | 3.209 | 1.558 | up |
|  | 3-Hydroxyglutaric acid | Organic acids | 1177.556 | 1.055 | up |
|  | Isocitric Acid* | Organic acids | 2.553 | 1.856 | up |
|  | 2-Hydroxyhexadecanoic acid | Organic acids | 2.653 | 1.251 | up |
|  | 10-Hydroxydecanoic acid | Lipids | 2.242 | 1.127 | up |
|  | Oleamide (9-Octadecenamide) | Lipids | 2.476 | 2.113 | up |
|  | 17-Hydroxylinolenic acid | Lipids | 2.267 | 2.065 | up |
|  | 9S-Hydroxy-10E,12Z-octadecadienoic acid | Lipids | 2.612 | 1.712 | up |
|  | 13-Hydroxy-9Z,11E-octadecadienoic acid | Lipids | 25.716 | 1.745 | up |
|  | 12,13-DHOME; (9Z)-12,13-Dihydroxyoctadec-9-enoic acid | Lipids | 2.088 | 1.813 | up |
|  | 2-Linoleoylglycerol* | Lipids | 5.365 | 1.729 | up |
|  | LysoPE 15:0(2n isomer)* | Lipids | 2.612 | 1.701 | up |
|  | LysoPE 15:0* | Lipids | 2.666 | 1.708 | up |
|  | LysoPE 18:1(2n isomer)* | Lipids | 2.106 | 1.325 | up |
|  | LysoPE 18:1* | Lipids | 2.345 | 1.431 | up |
|  | LysoPC 15:0* | Lipids | 4.805 | 1.694 | up |
|  | LysoPE 18:0(2n isomer)* | Lipids | 2.230 | 1.384 | up |
|  | LysoPC 15:0(2n isomer)* | Lipids | 248.969 | 1.570 | up |
|  | LysoPE 18:0* | Lipids | 2.333 | 1.418 | up |
|  | LysoPG 16:0 | Lipids | 2.628 | 1.368 | up |
|  | LysoPE 20:3* | Lipids | 3.612 | 1.649 | up |
|  | LysoPE 20:2* | Lipids | 2.169 | 1.279 | up |
|  | LysoPE 20:2(2n isomer)* | Lipids | 2.929 | 1.366 | up |
|  | LysoPC 17:0(2n isomer)* | Lipids | 211.923 | 1.555 | up |
|  | LysoPC 19:0 | Lipids | 4.879 | 1.639 | up |
|  | LysoPC 20:5 | Lipids | 2.637 | 1.517 | up |
|  | N-Acetyl-L-Glutamine | Amino acids and derivatives | 0.404 | 1.564 | down |
|  | N-Acetyl-DL-phenylalanine | Amino acids and derivatives | 0.444 | 1.305 | down |
|  | 3-hydroxyphenylacetic acid | Phenolic acids | 0.000 | 1.564 | down |
|  | Caffeic acid | Phenolic acids | 0.000 | 1.568 | down |

|  | Orsellinic acid ethyl ester | Phenolic acids | 0.423 | 1.532 | down |
| --- | --- | --- | --- | --- | --- |
|  | Feruloyl syringic acid | Phenolic acids | 0.330 | 1.336 | down |
|  | Salireposide | Phenolic acids | 0.471 | 1.035 | down |
|  | Uracil | Nucleotides and derivatives | 0.231 | 2.012 | down |
|  | Guanine* | Nucleotides and derivatives | 0.209 | 1.285 | down |
|  | Isoguanine* | Nucleotides and derivatives | 0.286 | 1.725 | down |
|  | Cordycepin (3'-Deoxyadenosine)* | Nucleotides and derivatives | 0.001 | 2.260 | down |
|  | 2'-Deoxyadenosine* | Nucleotides and derivatives | 0.273 | 2.115 | down |
|  | 2'-Deoxyguanosine | Nucleotides and derivatives | 0.309 | 2.204 | down |
|  | Crotonoside | Nucleotides and derivatives | 0.476 | 1.364 | down |
|  | N6-(2-Hydroxyethyl)adenosine | Nucleotides and derivatives | 0.117 | 1.776 | down |
|  | 2-(Dimethylamino)guanosine | Nucleotides and derivatives | 0.286 | 1.643 | down |
|  | Chrysoeriol | Flavonoids | 0.345 | 1.062 | down |
|  | Rhamnocitrin (7-Methylkaempferol) | Flavonoids | 0.142 | 1.620 | down |
|  | Diosmetin-7-O-rutinoside (Diosmin) | Flavonoids | 0.191 | 1.648 | down |
|  | Bergaptol | Lignans and Coumarins | 0.217 | 1.637 | down |
|  | 3-Methyl-1-pentanol | Others | 0.437 | 1.264 | down |
|  | D-Glucono-1,5-lactone | Others | 0.001 | 2.262 | down |
|  | D-Galactaric acid | Others | 0.463 | 1.075 | down |
|  | Oxalic acid | Organic acids | 0.003 | 1.609 | down |
|  | Hydroxypyruvic acid | Organic acids | 0.001 | 1.437 | down |
|  | succinic acid | Organic acids | 0.000 | 1.571 | down |
|  | β-Hydroxyisovaleric acid | Organic acids | 0.364 | 1.078 | down |
|  | Methylenesuccinic acid | Organic acids | 0.432 | 1.491 | down |
|  | 4-Hydroxy-2-Oxopentanoic Acid | Organic acids | 0.000 | 1.546 | down |
|  | 4-Hydroxycyclohexylcarboxylic acid | Organic acids | 0.014 | 1.049 | down |
|  | α-Ketoglutaric acid | Organic acids | 0.001 | 1.056 | down |
|  | cis-Citral | Organic acids | 0.025 | 1.056 | down |
|  | 5-Acetamidopentanoic Acid | Organic acids | 0.001 | 1.570 | down |
|  | Triethyl citrate | Organic acids | 0.370 | 1.951 | down |
|  | 2-Dodecenedioic acid | Lipids | 0.001 | 2.231 | down |
|  | 1-O-Caffeoylglycerol | Lipids | 0.001 | 1.607 | down |
|  | 1-Eicosanol | Lipids | 0.170 | 1.326 | down |
|  | 9-Hydroperoxy-10E,12,15Z-octadecatrienoic acid | Lipids | 0.255 | 2.067 | down |
|  | 13S-Hydroperoxy-9Z,11E-octadecadienoic acid | Lipids | 0.001 | 1.571 | down |
|  | 9-Hydroperoxy-9Z,11E-Octadecadienoic Acid | Lipids | 0.400 | 1.472 | down |
|  | 2-α-Linolenoyl-glycerol* | Lipids | 0.425 | 1.515 | down |
|  | LysoPC 18:3(2n isomer)* | Lipids | 0.460 | 1.936 | down |
| MC vs MW | Phenol | Phenolic acids | 3.438 | 1.135 | up |
|  | Phenyl acetate | Phenolic acids | 3.068 | 1.030 | up |
|  | 2-Methylbenzoic Acid | Phenolic acids | 2027.370 | 1.016 | up |
|  | 3,4-Dimethylbenzoic Acid | Phenolic acids | 2.512 | 1.222 | up |
|  | Methyl Anthranilate | Phenolic acids | 871.481 | 1.410 | up |
|  | Phenoxyacetic acid | Phenolic acids | 43.861 | 1.223 | up |
|  | 3-AminoSalicylic acid | Phenolic acids | 4137.370 | 1.036 | up |
|  | Propylparaben | Phenolic acids | 3.606 | 1.160 | up |
|  | Syringaldehyde; 4-Hydroxy-3,5-Dimethoxybenzaldehyde | Phenolic acids | 2.334 | 1.133 | up |
|  | 2,4-Dinitrophenol | Phenolic acids | 3.651 | 1.293 | up |
|  | Methyl ferulate | Phenolic acids | 2.377 | 1.202 | up |
|  | Ferulic acid methyl ester | Phenolic acids | 3.557 | 1.336 | up |
|  | Vanillyl Butyl Ether | Phenolic acids | 1564.074 | 1.429 | up |
|  | Benzoyltartaric acid | Phenolic acids | 18.251 | 1.319 | up |
|  | 6-O-Glucosyl-caffeoylbenzoic Acid | Phenolic acids | 6400.000 | 1.424 | up |
|  | Uridine | Nucleotides and derivatives | 832.593 | 1.428 | up |
|  | 1-beta-D-Arabinofuranosylurac il | Nucleotides and derivatives | 2.021 | 1.193 | up |
|  | Inosine | Nucleotides and derivatives | 4.052 | 1.374 | up |
|  | 9-(Arabinosyl)hypoxanthine | Nucleotides and derivatives | 13.303 | 1.134 | up |
|  | N6-methyladenosine | Nucleotides and derivatives | 3185.185 | 1.420 | up |
|  | 5-Aminoimidazole ribonucleotide | Nucleotides and derivatives | 2.301 | 1.245 | up |
|  | 2'-Deoxyinosine-5'-monophosphate | Nucleotides and derivatives | 65.593 | 1.384 | up |
|  | Matairesinol | Lignans and Coumarins | 3.218 | 1.284 | up |
|  | Arctigenin | Lignans and Coumarins | 11.019 | 1.387 | up |
|  | Nicotinamide | Others | 2.736 | 1.077 | up |
|  | D-Threitol | Others | 5.334 | 1.345 | up |
|  | Lactitol | Others | 54177.778 | 1.406 | up |
|  | Diferuloylcadaverine | Alkaloids | 15296.630 | 1.031 | up |
|  | 1-Oxo-Siaresinolic acid | Terpenoids | 2.575 | 1.312 | up |
|  | 2α,3α,19α,23-tetrahydroxy-12-ursen-28-oic acid | Terpenoids | 2.069 | 1.029 | up |
|  | 2α,3β,19α,23-Tetrahydroxyolean-12-en-28-oic acid | Terpenoids | 2.186 | 1.069 | up |
|  | 1β,2α,3α,19α,23-Pentahydroxyurs-12-en-28-oic acid | Terpenoids | 7.394 | 1.158 | up |
|  | Methanesulfonic acid | Organic acids | 4.475 | 1.087 | up |
|  | 3-Hydroxybutyric acid | Organic acids | 2.123 | 1.370 | up |
|  | Glutaric acid | Organic acids | 2.161 | 1.247 | up |
|  | 2-Hydroxyphenylacetic acid | Organic acids | 3.298 | 1.133 | up |
|  | D-(-)-Mandelic acid | Organic acids | 10.296 | 1.152 | up |
|  | Decanoic acid | Organic acids | 3.336 | 1.039 | up |
|  | 1-Hydroxy-2-Naphthoate | Organic acids | 2.268 | 1.322 | up |
|  | Undecylic Acid | Lipids | 3.244 | 1.115 | up |

| 10-Hydroxydecanoic acid | Lipids | 2.342 | 1.226 | up |
| --- | --- | --- | --- | --- |
| Dodecanoic acid (Lauric acid) | Lipids | 4.313 | 1.240 | up |
| Tridecanoic Acid | Lipids | 3.041 | 1.209 | up |
| 12-Methyltetradecanoic Acid | Lipids | 5.829 | 1.214 | up |
| Oleic acid | Lipids | 12.144 | 1.166 | up |
| 13(s)-hydroperoxy-(9z,11e,15z)-octadecatrienoic acid | Lipids | 2.950 | 1.159 | up |
| LysoPC 19:0 | Lipids | 3.867 | 1.292 | up |
| N,N-Dimethylglycine | Amino acids and derivatives | 0.000 | 1.433 | down |
| L-Proline | Amino acids and derivatives | 0.020 | 1.058 | down |
| Cycloleucine | Amino acids and derivatives | 0.064 | 1.385 | down |
| D-Proline betaine | Amino acids and derivatives | 0.480 | 1.126 | down |
| L-Methionine | Amino acids and derivatives | 0.001 | 1.432 | down |
| L-Phenylalanine | Amino acids and derivatives | 0.255 | 1.388 | down |
| N-Acetyl-L-leucine | Amino acids and derivatives | 0.300 | 1.084 | down |
| L-Tyrosine | Amino acids and derivatives | 0.210 | 1.319 | down |
| Cyclo(Ser-Pro) | Amino acids and derivatives | 0.389 | 1.418 | down |
| N6-Acetyl-L-lysine | Amino acids and derivatives | 0.166 | 1.287 | down |
| L-Glycyl-L-isoleucine* | Amino acids and derivatives | 0.001 | 1.054 | down |
| N-Glycyl-L-leucine* | Amino acids and derivatives | 0.001 | 1.059 | down |
| Trimethyllysine | Amino acids and derivatives | 0.175 | 1.348 | down |
| L-Homocitrulline | Amino acids and derivatives | 0.000 | 1.436 | down |
| Cyclo(Pro-Val) | Amino acids and derivatives | 0.233 | 1.057 | down |
| L-Tryptophan | Amino acids and derivatives | 0.082 | 1.085 | down |
| cinnamoylglycine | Amino acids and derivatives | 0.000 | 1.434 | down |
| N-Acetyl-L-phenylalanine | Amino acids and derivatives | 0.058 | 1.151 | down |
| N-Acetyl-DL-phenylalanine | Amino acids and derivatives | 0.204 | 1.043 | down |
| Cyclo(Pro-Leu) | Amino acids and derivatives | 0.257 | 1.260 | down |
| N-Acetyl-L-Arginine | Amino acids and derivatives | 0.124 | 1.112 | down |
| N'-Formylkynurenine | Amino acids and derivatives | 0.034 | 1.198 | down |
| Cyclo(D-Phe-L-Pro) | Amino acids and derivatives | 0.331 | 1.124 | down |
| Cyclo(Pro-Phe) | Amino acids and derivatives | 0.331 | 1.072 | down |
| N-(3-Indolylacetyl)-L-alanine | Amino acids and derivatives | 0.106 | 1.230 | down |
| N-Acetyl-L-Tryptophan | Amino acids and derivatives | 0.000 | 1.435 | down |
| L-γ-Glutamyl-L-leucine | Amino acids and derivatives | 0.145 | 1.427 | down |
| Tyrosol | Phenolic acids | 0.370 | 1.250 | down |
| 2,3-Dihydroxybenzoic Acid* | Phenolic acids | 0.125 | 1.362 | down |
| 2,5-Dihydroxybenzoic Acid; Gentisic Acid* | Phenolic acids | 0.119 | 1.366 | down |
| 3,4-Dihydroxybenzoic Acid (Protocatechuic acid)* | Phenolic acids | 0.127 | 1.365 | down |
| Ethylparaben | Phenolic acids | 0.167 | 1.359 | down |
| Coniferaldehyde | Phenolic acids | 0.158 | 1.345 | down |
| Caffeic acid | Phenolic acids | 0.009 | 1.064 | down |
| coniferyl alcohol | Phenolic acids | 0.031 | 1.026 | down |
| Hydroxyphenyllactic acid | Phenolic acids | 0.114 | 1.312 | down |
| Methyl 2,4-dihydroxyphenylacetate | Phenolic acids | 0.101 | 1.326 | down |
| Homovanillic acid; 4-Hydroxy-3-methoxyphenylacetic acid | Phenolic acids | 0.000 | 1.396 | down |
| Methyl caffeate | Phenolic acids | 0.000 | 1.435 | down |
| Sinapinaldehyde | Phenolic acids | 0.105 | 1.115 | down |
| Arbutin | Phenolic acids | 0.000 | 1.437 | down |
| 1-O-Salicyl-D-glucose | Phenolic acids | 0.308 | 1.160 | down |
| Glucosyloxybenzoic Acid | Phenolic acids | 0.307 | 1.135 | down |
| 3-Methoxy-4-hydroxyphenol-1-O-β-D-glucoside (Tachioside) | Phenolic acids | 0.000 | 1.436 | down |
| 3-Hydroxy-4-isopropylbenzylalcohol-3-O-glucoside | Phenolic acids | 0.061 | 1.287 | down |
| 3,4,5-Trimethoxyphenyl-1-O-Glucoside | Phenolic acids | 0.145 | 1.149 | down |
| Gallic acid-4-O-glucoside | Phenolic acids | 0.000 | 1.433 | down |
| 2-(3,4-dihydroxyphenyl)ethanediol 1-O-β-D-glucopyranoside | Phenolic acids | 0.000 | 1.429 | down |
| Koaburaside | Phenolic acids | 0.201 | 1.300 | down |
| 3-O-p-Coumaroylquinic acid* | Phenolic acids | 0.158 | 1.131 | down |
| P-Methoyxcinnamate glucoside | Phenolic acids | 0.000 | 1.434 | down |
| 6-O-Caffeoyl-D-glucose | Phenolic acids | 0.295 | 1.194 | down |
| coniferyl alcohol-4-O-glucoside (Coniferin) | Phenolic acids | 0.283 | 1.182 | down |
| Syringaldehyde-4-O-glucoside | Phenolic acids | 0.000 | 1.426 | down |
| 1-O-Feruloyl-D-Glucose | Phenolic acids | 0.020 | 1.139 | down |
| Chlorogenic acid methyl ester | Phenolic acids | 0.196 | 1.012 | down |
| Trihydroxycinnamoylquinic acid | Phenolic acids | 0.114 | 1.297 | down |
| Feruloyl syringic acid | Phenolic acids | 0.270 | 1.238 | down |
| 6-O-Glucosyl-feruloylbenzoic Acid | Phenolic acids | 0.143 | 1.318 | down |
| p-Coumaroylferuloyltartaric acid | Phenolic acids | 0.201 | 1.081 | down |
| Isochlorogenic acid A* | Phenolic acids | 0.023 | 1.262 | down |
| 3,5-Dicaffeoylquinic acid* | Phenolic acids | 0.000 | 1.038 | down |
| Isochlorogenic acid B* | Phenolic acids | 0.021 | 1.282 | down |
| Rosmarinic acid-3'-O-glucoside | Phenolic acids | 0.000 | 1.431 | down |
| 3,5-O-Dicaffeoylquinic acid Methyl Ester | Phenolic acids | 0.056 | 1.096 | down |
| 7-Methylguanine | Nucleotides and derivatives | 0.213 | 1.244 | down |
| 6-Methylmercaptopurine | Nucleotides and derivatives | 0.211 | 1.407 | down |
| 2'-O-Methyladenosine | Nucleotides and derivatives | 0.121 | 1.316 | down |
| Xanthosine | Nucleotides and derivatives | 0.016 | 1.229 | down |
| N6-(2-Hydroxyethyl)adenosine | Nucleotides and derivatives | 0.403 | 1.335 | down |
| 2-(Dimethylamino)guanosine | Nucleotides and derivatives | 0.334 | 1.334 | down |

| Succinyladenosine | Nucleotides and derivatives | 0.001 | 1.429 | down |
| --- | --- | --- | --- | --- |
| Ribosyladenosine | Nucleotides and derivatives | 0.149 | 1.290 | down |
| Uridine 5'-diphosphate | Nucleotides and derivatives | 0.000 | 1.434 | down |
| Nicotinic acid adenine dinucleotide | Nucleotides and derivatives | 0.001 | 1.423 | down |
| Pinocembrin (Dihydrochrysin) | Flavonoids | 0.000 | 1.435 | down |
| 5-Hydroxy-7-methoxyflavone | Flavonoids | 0.000 | 1.429 | down |
| Naringenin (5,7,4'-Trihydroxyflavanone) | Flavonoids | 0.046 | 1.122 | down |
| Eriodictyol (5,7,3',4'-Tetrahydroxyflavanone) | Flavonoids | 0.000 | 1.430 | down |
| Quercetin-3-O-Sulfonate | Flavonoids | 0.177 | 1.296 | down |
| Quercetin-3-O-glucoside (Isoquercitrin) | Flavonoids | 0.000 | 1.427 | down |
| Luteolin-7-O-neohesperidoside (lonicerin) | Flavonoids | 0.000 | 1.359 | down |
| Quercetin-3-O-(4''-O-glucosyl)rhamnoside | Flavonoids | 0.024 | 1.368 | down |
| 6,7-Dihydroxy-4-methylcoumarin | Lignans and Coumarins | 0.442 | 1.132 | down |
| Bergaptol | Lignans and Coumarins | 0.174 | 1.376 | down |
| scopoletin-7-O-glucoside (Scopolin) | Lignans and Coumarins | 0.197 | 1.066 | down |
| scopoletin-7-O-glucuronide | Lignans and Coumarins | 0.155 | 1.062 | down |
| Dihydrodehydrodiconiferyl alcohol-4-O-glucoside | Lignans and Coumarins | 0.149 | 1.156 | down |
| lariciresinol-4'-O-glucoside | Lignans and Coumarins | 0.000 | 1.432 | down |
| Isonicotinic acid* | Others | 0.080 | 1.380 | down |
| Nicotinic acid (Vitamin B3)* | Others | 0.073 | 1.388 | down |
| 2-Dehydro-3-deoxy-L-arabinonate | Others | 0.185 | 1.108 | down |
| D-Arabinose* | Others | 0.157 | 1.329 | down |
| 1,6-anhydro-β-D-glucose | Others | 0.000 | 1.435 | down |
| Norepinephrine | Others | 0.442 | 1.132 | down |
| Pyridoxine | Others | 0.281 | 1.260 | down |
| 3,7-Dihydroxychromen-4-one | Others | 0.284 | 1.047 | down |
| D-Glucono-1,5-lactone | Others | 0.072 | 1.153 | down |
| D-Galactose* | Others | 0.012 | 1.392 | down |
| D-Fructose* | Others | 0.038 | 1.413 | down |
| D-Glucose* | Others | 0.039 | 1.423 | down |
| D-Mannose* | Others | 0.010 | 1.064 | down |
| D-Erythrose-4-phosphate | Others | 0.150 | 1.101 | down |
| D-Galactaric acid | Others | 0.019 | 1.177 | down |
| D-Pantothenic Acid | Others | 0.130 | 1.399 | down |
| Nicotinate D-ribonucleoside | Others | 0.395 | 1.287 | down |
| 1-(sn-Glycero-3-phospho)-1D-myo-inositol | Others | 0.000 | 1.430 | down |
| Melibiose* | Others | 0.154 | 1.337 | down |
| D-Maltose* | Others | 0.077 | 1.264 | down |
| D-Trehalose* | Others | 0.313 | 1.406 | down |
| D-Sucrose* | Others | 0.315 | 1.355 | down |
| Isomaltulose* | Others | 0.096 | 1.352 | down |
| Maltotriose* | Others | 0.183 | 1.200 | down |
| D-Panose* | Others | 0.000 | 1.435 | down |
| Raffinose* | Others | 0.104 | 1.365 | down |
| Dehydrodiconiferyl alcohol-4-O-glucoside | Others | 0.028 | 1.314 | down |
| D(+)-Melezitose O-rhamnoside | Others | 0.215 | 1.056 | down |
| Stachyose* | Others | 0.000 | 1.435 | down |
| D-Maltotetraose* | Others | 0.097 | 1.028 | down |
| Choline | Alkaloids | 0.008 | 1.431 | down |
| N-Benzylmethylene isomethylamine | Alkaloids | 0.286 | 1.408 | down |
| Phenethylamine | Alkaloids | 0.000 | 1.433 | down |
| N-Acetylcadaverine | Alkaloids | 0.000 | 1.038 | down |
| 4-Hydroxymandelonitrile | Alkaloids | 0.389 | 1.069 | down |
| N-(4-Aminobutyl)benzamide | Alkaloids | 0.423 | 1.199 | down |
| Spermine | Alkaloids | 0.474 | 1.080 | down |
| N-Oleoylethanolamine | Alkaloids | 0.216 | 1.346 | down |
| N'-p-Coumaroylagmatine-glucoside | Alkaloids | 0.316 | 1.011 | down |
| Blumenol C | Terpenoids | 0.133 | 1.388 | down |
| L-Lactic Acid | Organic acids | 0.053 | 1.109 | down |
| Succinic anhydride | Organic acids | 0.107 | 1.102 | down |
| 2-Aminoisobutyric acid | Organic acids | 0.012 | 1.430 | down |
| 5-Aminovaleric acid | Organic acids | 0.110 | 1.128 | down |
| succinic acid | Organic acids | 0.273 | 1.044 | down |
| Aminomalonic acid | Organic acids | 0.267 | 1.047 | down |
| 2-Picolinic acid | Organic acids | 0.165 | 1.294 | down |
| Pipecolic acid | Organic acids | 0.061 | 1.361 | down |
| Citraconic acid | Organic acids | 0.337 | 1.273 | down |
| Methylenesuccinic acid | Organic acids | 0.000 | 1.427 | down |
| 2-Hydroxyisocaproic acid | Organic acids | 0.144 | 1.408 | down |
| 2-Hydroxy-4-methylpentanoic acid | Organic acids | 0.137 | 1.412 | down |
| 1-Methylpiperidine-2-carboxylic acid | Organic acids | 0.405 | 1.191 | down |
| L-Citramalic acid | Organic acids | 0.060 | 1.292 | down |
| 2-Hydroxyglutaric Acid | Organic acids | 0.231 | 1.059 | down |
| cis-Citral | Organic acids | 0.498 | 1.030 | down |
| 2-Isopropylmalic Acid* | Organic acids | 0.073 | 1.337 | down |
| 2-Propylmalic Acid* | Organic acids | 0.066 | 1.343 | down |
| Isocitric Acid* | Organic acids | 0.018 | 1.347 | down |
| Citric Acid* | Organic acids | 0.028 | 1.279 | down |

| Quinic Acid | Organic acids | 0.098 | 1.153 | down |
| --- | --- | --- | --- | --- |
| 4,8-Dihydroxyquinoline-2-carboxylic acid | Organic acids | 0.113 | 1.197 | down |
| Jasmonic acid | Organic acids | 0.000 | 1.434 | down |
| 2-Hydroxyhexadecanoic acid | Organic acids | 0.318 | 1.343 | down |
| 10-Heptadecenoic Acid | Lipids | 0.239 | 1.346 | down |
| α-Linolenic Acid* | Lipids | 0.123 | 1.409 | down |
| γ-Linolenic Acid* | Lipids | 0.144 | 1.379 | down |
| Linoleic acid | Lipids | 0.137 | 1.420 | down |
| 2R-hydroxy-9Z,12Z,15Z-octadecatrienoic acid | Lipids | 0.130 | 1.297 | down |
| 17-Hydroxylinolenic acid | Lipids | 0.197 | 1.318 | down |
| 13S-Hydroxy-9Z,11E,15Z-octadecatrienoic acid | Lipids | 0.288 | 1.310 | down |
| 9S-Hydroxy-10E,12Z-octadecadienoic acid | Lipids | 0.342 | 1.290 | down |
| 9(10)-EpOME;(9R,10S)-(12Z)-9,10-Epoxyoctadecenoic acid | Lipids | 0.444 | 1.215 | down |
| 13(S)-HODE;13(S)-Hydroxyoctadeca-9Z,11E-dienoic acid | Lipids | 0.308 | 1.293 | down |
| 3-Dehydrosphinganine | Lipids | 0.179 | 1.252 | down |
| 4-Hydroxysphinganine | Lipids | 0.070 | 1.402 | down |
| 12-Oxo-5,8,10,14-eicosatetraenoic acid | Lipids | 0.004 | 1.038 | down |
| 2-α-Linolenoyl-glycerol* | Lipids | 0.062 | 1.430 | down |
| 1-α-Linolenoyl-glycerol* | Lipids | 0.112 | 1.435 | down |
| 2-Linoleoylglycerol* | Lipids | 0.054 | 1.424 | down |
| 1-Oleoyl-Sn-Glycerol | Lipids | 0.191 | 1.412 | down |
| LysoPE 14:0* | Lipids | 0.090 | 1.421 | down |
| LysoPE 14:0(2n isomer)* | Lipids | 0.057 | 1.425 | down |
| LysoPE 15:1* | Lipids | 0.226 | 1.274 | down |
| LysoPE 15:1(2n isomer)* | Lipids | 0.178 | 1.268 | down |
| LysoPE 15:0(2n isomer)* | Lipids | 0.184 | 1.409 | down |
| LysoPE 15:0* | Lipids | 0.258 | 1.367 | down |
| LysoPE 16:3 | Lipids | 0.002 | 1.432 | down |
| LysoPE 16:1* | Lipids | 0.192 | 1.392 | down |
| LysoPE 16:1(2n isomer)* | Lipids | 0.098 | 1.413 | down |
| LysoPE 16:0(2n isomer)* | Lipids | 0.148 | 1.366 | down |
| LysoPE 16:0* | Lipids | 0.140 | 1.371 | down |
| LysoPE 17:1(2n isomer)* | Lipids | 0.035 | 1.408 | down |
| LysoPE 17:1* | Lipids | 0.049 | 1.383 | down |
| LysoPC 14:0 | Lipids | 0.229 | 1.242 | down |
| LysoPE 18:3(2n isomer)* | Lipids | 0.056 | 1.374 | down |
| LysoPE 18:3* | Lipids | 0.078 | 1.360 | down |
| LysoPE 18:2(2n isomer)* | Lipids | 0.055 | 1.388 | down |
| LysoPE 18:2* | Lipids | 0.078 | 1.366 | down |
| LysoPE 18:1(2n isomer)* | Lipids | 0.123 | 1.423 | down |
| LysoPE 18:1* | Lipids | 0.169 | 1.416 | down |
| LysoPE 18:0(2n isomer)* | Lipids | 0.145 | 1.415 | down |
| LysoPC 15:0(2n isomer)* | Lipids | 0.341 | 1.165 | down |
| LysoPE 18:0* | Lipids | 0.276 | 1.332 | down |
| LysoPG 16:0 | Lipids | 0.479 | 1.244 | down |
| LysoPC 16:2(2n isomer)* | Lipids | 0.260 | 1.421 | down |
| LysoPC 16:1(2n isomer)* | Lipids | 0.255 | 1.418 | down |
| LysoPC 16:0(2n isomer)* | Lipids | 0.188 | 1.406 | down |
| LysoPC 16:0* | Lipids | 0.257 | 1.389 | down |
| LysoPE 20:5 | Lipids | 0.095 | 1.409 | down |
| LysoPE 20:4(2n isomer)* | Lipids | 0.076 | 1.413 | down |
| LysoPE 20:4* | Lipids | 0.088 | 1.403 | down |
| LysoPE 20:3(2n isomer)* | Lipids | 0.055 | 1.408 | down |
| LysoPE 20:3* | Lipids | 0.084 | 1.393 | down |
| LysoPC 17:2 | Lipids | 0.136 | 1.388 | down |
| LysoPE 20:2* | Lipids | 0.121 | 1.419 | down |
| LysoPE 20:2(2n isomer)* | Lipids | 0.001 | 1.436 | down |
| LysoPC 17:1 | Lipids | 0.437 | 1.002 | down |
| LysoPC 17:0* | Lipids | 0.381 | 1.294 | down |
| LysoPC 17:0(2n isomer)* | Lipids | 0.217 | 1.360 | down |
| LysoPC 18:4 | Lipids | 0.081 | 1.399 | down |
| LysoPC 18:3(2n isomer)* | Lipids | 0.058 | 1.402 | down |
| LysoPC 18:3* | Lipids | 0.143 | 1.422 | down |
| LysoPC 18:2* | Lipids | 0.152 | 1.386 | down |
| LysoPC 18:2(2n isomer)* | Lipids | 0.065 | 1.409 | down |
| LysoPC 18:1(2n isomer)* | Lipids | 0.474 | 1.163 | down |
| LysoPC 18:0(2n isomer)* | Lipids | 0.320 | 1.375 | down |
| LysoPC 19:1 | Lipids | 0.229 | 1.349 | down |
| LysoPC 20:5 | Lipids | 0.080 | 1.376 | down |
| LysoPC 20:4 | Lipids | 0.115 | 1.404 | down |
| LysoPC 20:3 | Lipids | 0.080 | 1.384 | down |
| LysoPC 20:2(2n isomer)* | Lipids | 0.124 | 1.433 | down |
| LysoPC 20:2* | Lipids | 0.200 | 1.399 | down |
| LysoPC 22:6 | Lipids | 0.054 | 1.352 | down |
| LysoPC 22:5* | Lipids | 0.031 | 1.235 | down |
| LysoPC 22:5(2n isomer)* | Lipids | 0.001 | 1.433 | down |
| LysoPC 22:4 | Lipids | 0.040 | 1.242 | down |
| 1-Linolenoyl-rac-glycerol-diglucoside | Lipids | 0.072 | 1.381 | down |

|  | 1-Linoleoylglycerol-2,3-di-O-glucoside* | Lipids | 0.061 | 1.196 | down |
| --- | --- | --- | --- | --- | --- |
|  | 1-Linoleoyl-sn-glycerol-diglucoside | Lipids | 0.106 | 1.402 | down |
|  | 2-Linoleoylglycerol-1,3-di-O-glucoside* | Lipids | 0.058 | 1.199 | down |
| MW vs MWN | L-Alanine | Amino acids and derivatives | 13.741 | 1.365 | up |
|  | L-Lactic Acid | Organic acids | 17.134 | 1.309 | up |
|  | Succinic anhydride | Organic acids | 6.848 | 1.276 | up |
|  | 3-Methyl-1-pentanol | Others | 2.093 | 1.490 | up |
|  | 2-Aminoisobutyric acid | Organic acids | 58.915 | 1.654 | up |
|  | N,N-Dimethylglycine | Amino acids and derivatives | 10483.074 | 1.677 | up |
|  | Choline | Alkaloids | 83.725 | 1.660 | up |
|  | succinic acid | Organic acids | 2.715 | 1.208 | up |
|  | Aminomalonic acid | Organic acids | 2.640 | 1.206 | up |
|  | N-Benzylmethylene isomethylamine | Alkaloids | 2.034 | 1.429 | up |
|  | Phenethylamine | Alkaloids | 1467.074 | 1.136 | up |
|  | 2-Hydroxybenzaldehyde (Salicylaldehyde) | Phenolic acids | 3.909 | 1.361 | up |
|  | Isonicotinic acid* | Others | 4.211 | 1.559 | up |
|  | Nicotinic acid (Vitamin B3)* | Others | 4.463 | 1.570 | up |
|  | 2-Picolinic acid | Organic acids | 2.875 | 1.415 | up |
|  | 2-Hydroxyethylphosphonic acid | Organic acids | 2039.259 | 1.216 | up |
|  | Citraconic acid | Organic acids | 4.618 | 1.447 | up |
|  | Methylenesuccinic acid | Organic acids | 4209.593 | 1.669 | up |
|  | 2-Hydroxyisocaproic acid | Organic acids | 5.081 | 1.575 | up |
|  | 2-Hydroxy-4-methylpentanoic acid | Organic acids | 5.123 | 1.574 | up |
|  | L-Malic acid | Organic acids | 2.434 | 1.200 | up |
|  | 2,5-Dihydroxybenzaldehyde | Phenolic acids | 2.005 | 1.042 | up |
|  | Protocatechualdehyde | Phenolic acids | 2.108 | 1.066 | up |
|  | Salicylic acid | Phenolic acids | 2.053 | 1.056 | up |
|  | Tyrosol | Phenolic acids | 3.290 | 1.508 | up |
|  | L-Citramalic acid | Organic acids | 11.928 | 1.374 | up |
|  | 2-Dehydro-3-deoxy-L-arabinonate | Others | 6.455 | 1.251 | up |
|  | 2-Hydroxyglutaric Acid | Organic acids | 5.045 | 1.196 | up |
|  | L-Methionine | Amino acids and derivatives | 687.574 | 1.663 | up |
|  | D-Arabinose* | Others | 3.111 | 1.414 | up |
|  | Anisic acid | Phenolic acids | 2.256 | 1.365 | up |
|  | 2,3-Dihydroxybenzoic Acid* | Phenolic acids | 8.015 | 1.580 | up |
|  | 2,5-Dihydroxybenzoic Acid; Gentisic Acid* | Phenolic acids | 9.399 | 1.571 | up |
|  | 3,4-Dihydroxybenzoic Acid (Protocatechuic acid)* | Phenolic acids | 8.294 | 1.576 | up |
|  | 1,6-anhydro-β-D-glucose | Others | 5944.667 | 1.205 | up |
|  | Lumazine | Nucleotides and derivatives | 2274.333 | 1.680 | up |
|  | p-Coumaric acid | Phenolic acids | 2.350 | 1.038 | up |
|  | 7-Methylguanine | Nucleotides and derivatives | 3.750 | 1.367 | up |
|  | L-Phenylalanine | Amino acids and derivatives | 2.303 | 1.396 | up |
|  | 6-Methylmercaptopurine | Nucleotides and derivatives | 2.445 | 1.492 | up |
|  | Ethylparaben | Phenolic acids | 5.803 | 1.647 | up |
|  | 3-(4-Hydroxyphenyl)-propionic acid | Phenolic acids | 16.266 | 1.047 | up |
|  | 2,6-Dimethoxybenzaldehyde | Phenolic acids | 14.674 | 1.043 | up |
|  | 3,4-Dihydroxybenzeneacetic acid | Phenolic acids | 2.969 | 1.324 | up |
|  | Homogentisic acid | Phenolic acids | 2.900 | 1.267 | up |
|  | Norepinephrine | Others | 2.445 | 1.455 | up |
|  | Pyridoxine | Others | 3.079 | 1.507 | up |
|  | N-Acetyl-L-leucine | Amino acids and derivatives | 4.227 | 1.530 | up |
|  | 2-Isopropylmalic Acid* | Organic acids | 7.629 | 1.503 | up |
|  | 2-Propylmalic Acid* | Organic acids | 8.918 | 1.513 | up |
|  | Daphnetin | Lignans and Coumarins | 2.198 | 1.063 | up |
|  | 3,7-Dihydroxychromen-4-one | Others | 5.140 | 1.234 | up |
|  | Coniferaldehyde | Phenolic acids | 6.110 | 1.618 | up |
|  | p-Coumaric acid methyl ester | Phenolic acids | 2.260 | 1.279 | up |
|  | Caffeic acid | Phenolic acids | 87.066 | 1.153 | up |
|  | D-Galactose* | Others | 27.765 | 1.318 | up |
|  | D-Fructose* | Others | 8.214 | 1.378 | up |
|  | Inositol* | Others | 2.900 | 1.239 | up |
|  | D-Glucose* | Others | 7.448 | 1.400 | up |
|  | D-Mannose* | Others | 31.320 | 1.061 | up |
|  | coniferyl alcohol | Phenolic acids | 27.574 | 1.194 | up |
|  | L-Tyrosine | Amino acids and derivatives | 2.852 | 1.420 | up |
|  | Hydroxyphenyllactic acid | Phenolic acids | 9.270 | 1.532 | up |
|  | Methyl 2,4-dihydroxyphenylacetate | Phenolic acids | 9.693 | 1.540 | up |
|  | Homovanillic acid; 4-Hydroxy-3-methoxyphenylacetic acid | Phenolic acids | 389516.667 | 1.654 | up |
|  | N6-Acetyl-L-lysine | Amino acids and derivatives | 2.804 | 1.203 | up |
|  | Trimethyllysine | Amino acids and derivatives | 3.172 | 1.334 | up |
|  | L-Homocitrulline | Amino acids and derivatives | 1410.807 | 1.674 | up |
|  | Isocitric Acid* | Organic acids | 9.941 | 1.328 | up |
|  | Citric Acid* | Organic acids | 14.880 | 1.031 | up |
|  | Quinic Acid | Organic acids | 5.412 | 1.327 | up |
|  | p-Coumaric acid ethyl ester | Phenolic acids | 1261.826 | 1.209 | up |
|  | Ferulic acid* | Phenolic acids | 6.134 | 1.336 | up |
|  | Methyl caffeate | Phenolic acids | 1990.896 | 1.666 | up |
|  | Isoferulic Acid* | Phenolic acids | 5.895 | 1.349 | up |

| Dihydroferulic Acid | Phenolic acids | 2.945 | 1.178 | up |
| --- | --- | --- | --- | --- |
| 3,4-Dimethoxyphenyl acetic acid | Phenolic acids | 2.780 | 1.151 | up |
| Cyclo(Pro-Val) | Amino acids and derivatives | 3.895 | 1.223 | up |
| Orsellinic acid ethyl ester | Phenolic acids | 3.713 | 1.481 | up |
| Bergaptol | Lignans and Coumarins | 2.326 | 1.404 | up |
| L-Tryptophan | Amino acids and derivatives | 6.325 | 1.221 | up |
| 4,8-Dihydroxyquinoline-2-carboxylic acid | Organic acids | 7.317 | 1.187 | up |
| cinnamoylglycine | Amino acids and derivatives | 2665.444 | 1.674 | up |
| N-Acetyl-DL-phenylalanine | Amino acids and derivatives | 2.235 | 1.089 | up |
| Ethyl caffeate | Phenolic acids | 15.047 | 1.109 | up |
| D-Galactaric acid | Others | 44.361 | 1.331 | up |
| Blumenol C | Terpenoids | 3.872 | 1.445 | up |
| N-Acetyl-L-Arginine | Amino acids and derivatives | 6.476 | 1.273 | up |
| D-Pantothenic Acid | Others | 5.418 | 1.570 | up |
| N-Acetyl-L-tyrosine | Amino acids and derivatives | 3.215 | 1.387 | up |
| N'-Formylkynurenine | Amino acids and derivatives | 6.171 | 1.273 | up |
| β-Pseudouridine | Nucleotides and derivatives | 2.830 | 1.040 | up |
| Cyclo(Pro-Phe) | Amino acids and derivatives | 2.262 | 1.215 | up |
| N-(3-Indolylacetyl)-L-alanine | Amino acids and derivatives | 3.388 | 1.640 | up |
| N-Acetyl-L-Tryptophan | Amino acids and derivatives | 4965.222 | 1.679 | up |
| Pinocembrin (Dihydrochrysin) | Flavonoids | 2316.407 | 1.680 | up |
| Nicotinate D-ribonucleoside | Others | 2.391 | 1.514 | up |
| L-γ-Glutamyl-L-leucine | Amino acids and derivatives | 3.764 | 1.626 | up |
| 5-Hydroxy-7-methoxyflavone | Flavonoids | 315010.741 | 1.657 | up |
| Naringenin (5,7,4'-Trihydroxyflavanone) | Flavonoids | 11.566 | 1.276 | up |
| Arbutin | Phenolic acids | 5573.704 | 1.210 | up |
| 16-Hydroxyhexadecanoic acid | Lipids | 2.512 | 1.287 | up |
| α-Linolenic Acid* | Lipids | 2.325 | 1.586 | up |
| γ-Linolenic Acid* | Lipids | 2.069 | 1.433 | up |
| Linoleic acid | Lipids | 2.291 | 1.372 | up |
| 2'-O-Methyladenosine | Nucleotides and derivatives | 8.089 | 1.469 | up |
| Xanthosine | Nucleotides and derivatives | 6.280 | 1.550 | up |
| Eriodictyol (5,7,3',4'-Tetrahydroxyflavanone) | Flavonoids | 4586.519 | 1.677 | up |
| N7-Methylguanosine | Nucleotides and derivatives | 3.314 | 1.175 | up |
| 3-Dehydrosphinganine | Lipids | 2.853 | 1.189 | up |
| 1-O-Salicyl-D-glucose | Phenolic acids | 3.419 | 1.452 | up |
| Glucosyloxybenzoic Acid | Phenolic acids | 3.602 | 1.444 | up |
| 3-Methoxy-4-hydroxyphenol-1-O-β-D-glucoside (Tachioside) | Phenolic acids | 28456.296 | 1.680 | up |
| N6-(2-Hydroxyethyl)adenosine | Nucleotides and derivatives | 3.268 | 1.532 | up |
| 2-(Dimethylamino)guanosine | Nucleotides and derivatives | 2.983 | 1.514 | up |
| Protocatechuic acid-4-O-glucoside | Phenolic acids | 10.629 | 1.013 | up |
| 4-Hydroxysphinganine | Lipids | 3.689 | 1.574 | up |
| Myricetin | Flavonoids | 7.999 | 1.051 | up |
| N-Oleoylethanolamine | Alkaloids | 3.503 | 1.101 | up |
| 3,4,5-Trimethoxyphenyl-1-O-Glucoside | Phenolic acids | 4.868 | 1.251 | up |
| Gallic acid-4-O-glucoside | Phenolic acids | 7768.852 | 1.670 | up |
| 2-(3,4-dihydroxyphenyl)ethanediol 1-O-β-D-glucopyranoside | Phenolic acids | 7970.889 | 1.678 | up |
| Koaburaside | Phenolic acids | 7.366 | 1.480 | up |
| 1-(sn-Glycero-3-phospho)-1D-myo-inositol | Others | 2319.667 | 1.138 | up |
| Riboprine | Nucleotides and derivatives | 5.160 | 1.184 | up |
| 3-O-p-Coumaroylquinic acid* | Phenolic acids | 8.234 | 1.209 | up |
| P-Methoyxcinnamate glucoside | Phenolic acids | 24614.148 | 1.672 | up |
| 6-O-Caffeoyl-D-glucose | Phenolic acids | 2.017 | 1.265 | up |
| Melibiose* | Others | 5.355 | 1.563 | up |
| D-Maltose* | Others | 7.702 | 1.412 | up |
| D-Trehalose* | Others | 2.679 | 1.568 | up |
| D-Sucrose* | Others | 2.395 | 1.534 | up |
| Isomaltulose* | Others | 6.736 | 1.592 | up |
| Lactobiose* | Others | 9.338 | 1.066 | up |
| coniferyl alcohol-4-O-glucoside (Coniferin) | Phenolic acids | 2.175 | 1.193 | up |
| Syringaldehyde-4-O-glucoside | Phenolic acids | 3918.778 | 1.679 | up |
| Esculetin-7-O-quinic acid | Lignans and Coumarins | 11.677 | 1.041 | up |
| 5,6,15-Trihydroxy-7,9,11,13-eicosatetraenoic acid | Lipids | 2108.000 | 1.210 | up |
| 2-α-Linolenoyl-glycerol* | Lipids | 2.130 | 1.513 | up |
| 1-α-Linolenoyl-glycerol* | Lipids | 2.190 | 1.623 | up |
| Neochlorogenic acid (5-O-caffeoylquinic acid)* | Phenolic acids | 18.977 | 1.043 | up |
| Cryptochlorogenic acid (4-O-caffeoylquinic acid)* | Phenolic acids | 16.798 | 1.023 | up |
| Chlorogenic acid (3-O-caffeoylquinic acid)* | Phenolic acids | 19.073 | 1.042 | up |
| 2-Linoleoylglycerol* | Lipids | 2.004 | 1.443 | up |
| 1-O-Feruloyl-D-Glucose | Phenolic acids | 26.619 | 1.273 | up |
| Pinoresinol* | Lignans and Coumarins | 3.758 | 1.171 | up |
| Epipinoresinol* | Lignans and Coumarins | 3.730 | 1.155 | up |
| scopoletin-7-O-glucuronide | Lignans and Coumarins | 8.038 | 1.318 | up |
| Chlorogenic acid methyl ester | Phenolic acids | 8.858 | 1.347 | up |
| Trihydroxycinnamoylquinic acid | Phenolic acids | 4.894 | 1.614 | up |
| Feruloyl syringic acid | Phenolic acids | 3.108 | 1.311 | up |
| Riboflavin (Vitamin B2) | Others | 3.305 | 1.178 | up |
| Succinyladenosine | Nucleotides and derivatives | 262.354 | 1.624 | up |

| Ribosyladenosine | Nucleotides and derivatives | 6.741 | 1.228 | up |
| --- | --- | --- | --- | --- |
| Uridine 5'-diphosphate | Nucleotides and derivatives | 6935.556 | 1.210 | up |
| 2-Hydroxyphenol-1-O-glucosyl(6→1)rhamnoside | Phenolic acids | 9.173 | 1.059 | up |
| syringaresinol | Lignans and Coumarins | 2.254 | 1.237 | up |
| p-Coumaroylferuloylcadaverine | Alkaloids | 2.459 | 1.015 | up |
| LysoPE 14:0* | Lipids | 4.882 | 1.645 | up |
| LysoPE 14:0(2n isomer)* | Lipids | 5.390 | 1.633 | up |
| LysoPE 15:0(2n isomer)* | Lipids | 2.213 | 1.377 | up |
| LysoPE 15:0* | Lipids | 2.121 | 1.344 | up |
| 6-O-Glucosyl-caffeoylbenzoic Acid | Phenolic acids | 2.358 | 1.094 | up |
| LysoPE 16:3 | Lipids | 351.685 | 1.681 | up |
| 3',5,5',7-Tetrahydroxyflavanone-7-O-glucoside | Flavonoids | 3.359 | 1.408 | up |
| LysoPE 16:1* | Lipids | 2.516 | 1.523 | up |
| LysoPE 16:1(2n isomer)* | Lipids | 3.026 | 1.565 | up |
| LysoPE 16:0(2n isomer)* | Lipids | 2.658 | 1.413 | up |
| LysoPE 16:0* | Lipids | 2.782 | 1.437 | up |
| 6-O-Glucosyl-feruloylbenzoic Acid | Phenolic acids | 18.636 | 1.606 | up |
| LysoPE 17:1(2n isomer)* | Lipids | 7.640 | 1.607 | up |
| LysoPE 17:1* | Lipids | 7.884 | 1.570 | up |
| LysoPE 18:3(2n isomer)* | Lipids | 2.960 | 1.364 | up |
| LysoPE 18:3* | Lipids | 3.048 | 1.375 | up |
| LysoPE 18:2(2n isomer)* | Lipids | 2.752 | 1.377 | up |
| LysoPE 18:2* | Lipids | 2.720 | 1.348 | up |
| LysoPE 18:1(2n isomer)* | Lipids | 2.662 | 1.553 | up |
| LysoPE 18:1* | Lipids | 2.706 | 1.572 | up |
| LysoPE 18:0(2n isomer)* | Lipids | 2.295 | 1.519 | up |
| Quillaic acid | Terpenoids | 3.467 | 1.219 | up |
| Dicaffeoylshikimic acid | Phenolic acids | 15.330 | 1.068 | up |
| LysoPE 20:3(2n isomer)* | Lipids | 2.230 | 1.369 | up |
| LysoPE 20:3* | Lipids | 2.280 | 1.353 | up |
| Maltotriose* | Others | 2.882 | 1.223 | up |
| D-Panose* | Others | 13644.185 | 1.681 | up |
| D-Melezitose* | Others | 6.698 | 1.013 | up |
| Raffinose* | Others | 7.343 | 1.609 | up |
| LysoPE 20:2(2n isomer)* | Lipids | 292.785 | 1.680 | up |
| Isochlorogenic acid A* | Phenolic acids | 34.365 | 1.456 | up |
| 3,5-Dicaffeoylquinic acid* | Phenolic acids | 13612.519 | 1.673 | up |
| Isochlorogenic acid B* | Phenolic acids | 33.875 | 1.505 | up |
| 4,5-Dicaffeoylquinic acid* | Phenolic acids | 20.090 | 1.089 | up |
| 1,3-Dicaffeoylquinic acid* | Phenolic acids | 19.634 | 1.085 | up |
| Dehydrodiconiferyl alcohol-4-O-glucoside | Others | 14.753 | 1.424 | up |
| Dihydrodehydrodiconiferyl alcohol-4-O-glucoside | Lignans and Coumarins | 5.551 | 1.549 | up |
| 3,5-O-Dicaffeoylquinic acid Methyl Ester | Phenolic acids | 12.445 | 1.255 | up |
| LysoPC 22:5* | Lipids | 8.268 | 1.299 | up |
| LysoPC 22:4 | Lipids | 8.429 | 1.328 | up |
| Apigenin-6,8-di-C-glucoside (Vicenin-2) | Flavonoids | 3.627 | 1.170 | up |
| Quercetin-3-O-(4''-O-glucosyl)rhamnoside | Flavonoids | 2.233 | 1.039 | up |
| Nicotinic acid adenine dinucleotide | Nucleotides and derivatives | 867.519 | 1.207 | up |
| Stachyose* | Others | 10053.556 | 1.681 | up |
| D-Maltotetraose* | Others | 7.713 | 1.177 | up |
| Nystose* | Others | 10.130 | 1.091 | up |
| 3,4,5-Tricaffeoylquinic acid | Phenolic acids | 17.077 | 1.293 | up |
| 1-Linoleoylglycerol-2,3-di-O-glucoside* | Lipids | 3.781 | 1.233 | up |
| 2-Linoleoylglycerol-1,3-di-O-glucoside* | Lipids | 4.176 | 1.241 | up |
| N-Monomethyl-L-arginine | Amino acids and derivatives | 0.336 | 1.005 | down |
| cinnamic acid | Phenolic acids | 0.006 | 1.144 | down |
| Phenoxyacetic acid | Phenolic acids | 0.279 | 1.285 | down |
| 3-AminoSalicylic acid | Phenolic acids | 0.000 | 1.214 | down |
| Erianin | Phenolic acids | 0.465 | 1.286 | down |
| 1,7-Dimethylxanthine | Nucleotides and derivatives | 0.470 | 1.284 | down |
| Uridine | Nucleotides and derivatives | 0.167 | 1.280 | down |
| 9-(Arabinosyl)hypoxanthine | Nucleotides and derivatives | 0.305 | 1.073 | down |
| Apigenin-7-O-neohesperidoside (Rhoifolin) | Flavonoids | 0.001 | 1.669 | down |
| Diosmetin-7-O-rutinoside (Diosmin) | Flavonoids | 0.101 | 1.573 | down |
| 6-MethylCoumarin | Lignans and Coumarins | 0.000 | 1.201 | down |
| Arctigenin | Lignans and Coumarins | 0.309 | 1.440 | down |
| 2-(Acetylamino)-3-phenyl-2-propenoic acid | Alkaloids | 0.394 | 1.047 | down |
| 4-Hydroxycyclohexylcarboxylic acid | Organic acids | 0.009 | 1.026 | down |
| 2-Hydroxyphenylacetic acid | Organic acids | 0.405 | 1.117 | down |
| D-(-)-Mandelic acid | Organic acids | 0.435 | 1.293 | down |
| 2-Oxoheptanedionic acid | Organic acids | 0.184 | 1.276 | down |
| Lipoic acid | Organic acids | 0.339 | 1.128 | down |
| 10-Hydroxydecanoic acid | Lipids | 0.451 | 1.465 | down |
| 9,12-Octadecadien-6-Ynoic Acid | Lipids | 0.491 | 1.130 | down |
| 9,16-Dihydroxypalmitic acid | Lipids | 0.355 | 1.312 | down |
| 15(R)-Hydroxylinoleic Acid | Lipids | 0.283 | 1.027 | down |
